# Pruning the Tree: Comparing OTUs and ASVs in High‐Throughput Sequencing of 5S‐IGS Nuclear Ribosomal DNA in Phylogenetic Studies

**DOI:** 10.1002/ece3.72242

**Published:** 2025-10-07

**Authors:** Simone Cardoni, Roberta Piredda, Guido W. Grimm, Mariangela Santorsola, Ernst‐Detlef Schulze, Thomas Denk, Daniele De Luca, Marco Cosimo Simeone

**Affiliations:** ^1^ Water Research Institute (IRSA), National Research Council Taranto Italy; ^2^ Department of Research Infrastructures for Marine Biological Resources Naples Italy; ^3^ NBFC, National Biodiversity Future Center Palermo Italy; ^4^ Unaffiliated Orléans France; ^5^ Department of Biology and Biotechnology ‘Lazzaro Spallanzani’ University of Pavia Pavia Italy; ^6^ Max‐Planck Institute for Biogeochemistry Jena Germany; ^7^ Department of Palaeobiology Swedish Museum of Natural History Stockholm Sweden; ^8^ Department of Humanities Università Degli Studi Suor Orsola Benincasa Naples Italy; ^9^ Department of Agricultural and Forestry Science (DAFNE) Università Degli Studi Della Tuscia Viterbo Italy

**Keywords:** 5S nrDNA, DADA2, *Fagus*, high‐throughput sequencing, MOTHUR, phylogenetic analysis

## Abstract

Amplicon sequencing of the nuclear ribosomal 5S RNA gene arrays is highly promising for genotaxonomy, to delineate genetic resources of species and trace their evolution. However, the huge amount of data retrieved with this approach is difficult to manage and prone to redundancy, error, and computational difficulties. Reducing the amount of data per sample without losing molecular‐phylogenetic signal is therefore a crucial step for downstream analyses. In this work, we compared operational taxonomic units (OTUs, 100% identity) and amplicon sequence variants (ASVs) from 5S intergenic spacer (5S‐IGS) amplicons of seven beech species (*Fagus* spp.) obtained with two widely used and competing bioinformatics tools, MOTHUR and DADA2. We assessed quantitative (total number of sequences retrieved, proportion of sequences unique or shared across the different data sets) and qualitative (sample diversity, data ruggedness and congruence of the obtained phylogenies) differences among sample data profiles obtained with the two methods, and the capacity of the inferred phylogenies to capture diagnostic 5S‐IGS variant types. Over 70% of processed reads were shared between OTUs and ASVs. Despite a strong reduction (> 80%) of the representative sequences, DADA2‐ASVs identified all main 5S‐IGS variants known for *Fagus*, reflecting the phylogenetic, taxonomic and diversity patterns expected for each sample. In contrast, MOTHUR generated large proportions of rare OTUs and ASVs that complicated the obtained phylogenies and were inference‐wise redundant. We conclude that differences in the sequence variation detected by the two pipelines are minimal and do not result in different phylogenetic information. The more effective and computationally more efficient DADA2 ASVs may thus replace OTUs in future 5S‐IGS studies dealing with complex bioecological phenomena such as hybridisation, polyploidisation, drift and inferring evolutionary pathways of species systems, especially when using increasingly large sample sets.

## Graphical abstract

We compared the efficiency of operational taxonomic units (OTUs) and amplicon sequence variants (ASVs) of the 5S nrDNA arrays in evolutionary studies of a prominent tree genus of the Northern Hemisphere. Data targeting seven model samples of different beech (Fagus L., Fagaceae) species were processed in parallel with five different workflows implemented in the two most widely used and debated bioinformatics tools for HTS data inference and curation (Mothur and DADA2). The obtained results will allow the optimisation of the 5S‐HTS workflow for future studies on the assessment of phylogenetic/phylogeographic patterns in complex species systems, with emphasis on accuracy, easier manageability, interoperability and reusability of the produced data.

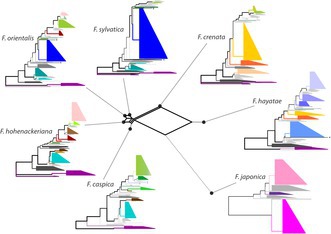

## 1 Introduction

The arrays encoding the 5S nuclear ribosomal RNA genes (5S nrDNA) are of considerable importance for biologists because they provide crucial information about genome organisation, molecular evolution, macro‐ and microevolutionary patterns and taxonomy (e.g., Ford and Southern 1973; Gilbert 1986; Forest and Bruneau 2000; Muir et al. 2000, 2001; Baum et al. 2001; Fulnecěk et al. 2002; Rooney and Ward 2005; Vierna et al. 2013; Simon et al. 2018; Garcia et al. 2020; Maiwald et al. 2024; Chen et al. 2024). In plants, the hundreds to thousands of 5S nrDNA repeat units per genome (Cloix et al. 2000; Torres‐Machorro et al. 2010) are separated by nontranscribed intergenic spacers (5S‐IGS) characterised by striking interindividual and intragenomic variability (Wang et al. 2023). Until recently, their composition and diversity could only be studied using work‐ and time‐intensive cloning and sequencing strategies (reviewed in Hemleben et al. 2021).

The advent of High‐Throughpute Squencing (HTS) approaches has tremendously simplified and increased the sequencing depth and variant resolution of the ribosomal gene arrays (e.g., Ganley and Kobayashi 2007). Typically, HTS data retrieved from nuclear‐encoded rDNA amplicon sequencing are used to assess the diversity of microorganisms in environmental samples (reviewed in Deiner et al. 2017; Ruppert et al. 2019). Yet, only a few studies have used HTS targeted sequencing of plant 5S nrDNA amplicons (5S‐HTS) for genotaxonomy or phylogenetic purposes (Piredda et al. 2021; Scoppola et al. 2022; Cardoni et al. 2022; Mandáková et al. 2024). These studies produced large and complex datasets with hundreds of thousands of sequence reads per sample covering both inter‐ and intra‐array diversity and providing evidence for lineage sorting, past hybridisation, introgression and ancient polyploidisation.

In our previous studies on Fagaceae (Piredda et al. 2021, oaks, genus *Quercus*; Cardoni et al. 2022, beeches, genus *Fagus*), we used the pre‐processing pipeline implemented in MOTHUR (Schloss et al. 2009) to identify and group identical HTS sequence reads into unique (representative) sequence variants, ‘Operational Taxonomic Units’ (OTUs), used for downstream analyses. The phylogenetic structure of the 5S nrDNA arrays in the target species had not previously been studied, except for some cloning projects anticipating large variability in oaks (Denk and Grimm 2010; Simeone et al. 2018; no data available for beeches). Since our aim was to capture all the existing genetic diversity in samples from defined biological sources (individuals, populations, species), we opted for OTUs with 100% sequence identity instead of using an artificial lower clustering threshold (generally 97% in standard biodiversity applications) to improve precision (Edgar 2018).

The amount of inferred OTUs (hundreds to thousands in each sample) is computationally challenging and therefore problematic for inferring phylogenies. Aside from the high number of tips compared to a relatively low number of distinct alignment patterns, tree inference and branch support estimation must accommodate signals from deep splits and terminal noise generated by ± stochastic mutational patterns comprised of large groups of near‐identical tips or sequence degradation (starting pseudogeny). Furthermore, the presence of numerous sequences occurring at low abundances may represent either relevant taxonomic/evolutionary signals or sequence artefacts (‘chimeras’) and contamination (Coissac et al. 2012; Antich et al. 2021; Gold et al. 2023).

In environmental biodiversity studies, classical morphological counts are generally compared to the retrieved OTUs to assess potential bias deriving from contamination and spurious sequencing (e.g., Nichols et al. 2018; Furlan et al. 2020; Škaloud et al. 2020; van der Loos and Nijland 2021). In our previous studies, no evidence of contamination or artefacts was found: using pooled DNA extracts from one to > 10 individuals of known forest species/stands, we observed consistent patterns of sequence variation well reflecting intra‐ and inter‐array variation at individual, specific and population levels (Piredda et al. 2021; Cardoni et al. 2022; Denk et al. 2024). In addition, in oaks, all abundant variants covered the same types of earlier broadly sampled clone data, and additional types showing mutational patterns expected for 5S‐IGS data (Piredda et al. 2021). In beeches, extremely rare and aberrant variants showed characteristic mutation patterns indicating beginning pseudogeny and silencing of arrays and were excluded using an abundance cut‐off (≥ 25 for first informative phylogenetic trees; ≥ 4 for deeper, sample‐wise analyses; Cardoni et al. 2022). Nevertheless, although the computation of phylogenetic trees and networks is now faster than ever (e.g., Kozlov et al. 2019; Piñeiro et al. 2020), large data manipulation and visualisation remains challenging (Theys et al. 2019; Palacios et al. 2021). Trees with 1000 or more tips can only be viewed in collapsed form as rooted phylograms (proportional branch lengths) or as radial cladogram (uninformative branch lengths, most common choice). They typically include many branches with low support and flat subtrees, and clades showing very little differentiation. Many similar or nearly identical tips inflate computation times during bootstrapping and final tree inference, while providing little additional information. On the other hand, since the abundances of obtained unique reads are not necessarily quantitative representations of their frequency in the 5S rDNA array, the reliance on abundance thresholds to reduce the set of variants to a reasonable tip set may be problematic as well.

In recent years, several algorithms have been developed to manage HTS sequencing errors and the amounts of generated data, such as ‘Amplicon Sequence Variants’ (ASVs) in DADA2 (Callahan et al. 2016). Such a strategy has been reported to be more accurate than the OTU clustering method to resolve ‘real’ sequence types differing by a single nucleotide and present in as few as two reads in a dataset. The way OTUs and ASVs are inferred differs in the two pipelines: OTUs are obtained through clustering algorithms collapsing sequences below a certain arbitrary or reference‐based similarity threshold. Within DADA2, ASVs are obtained through a machine‐learning approach calculating an error model based on the quality of the sequencing run, then applied to collapse sequences with errors.

Indeed, both OTUs and ASVs approaches have several inherent properties that make them desirable (Callahan et al. 2017; Schloss 2021). ASVs have been described as exchangeable across different studies, generally more reliable and produced in lower numbers than OTUs (e.g., Chiarello et al. 2022; Cholet et al. 2022), while OTUs appear more adequate to capture the community complexity, especially in terms of rare variants (Joos et al. 2020). Open questions remain about the influence of these algorithms on different sample types (e.g., real or mock communities), targeted amplicons (ribosomal genes, spacers or other functional genes), organisms and work objectives. Although several recent studies on environmental samples compared the performance of MOTHUR and DADA2 in the characterisation of taxonomic profiles (Tremblay and Yergeau 2019; Joos et al. 2020; Chiarello et al. 2022; Cholet et al. 2022; Liu et al. 2023), no previous study assessed their impact on phylogenetic reconstructions.

In this study, we contrasted the efficiency of OTUs and ASVs in evolutionary studies targeting the 5S nrDNA arrays. 5S‐HTS data from bulk DNA samples of seven beech species were processed in parallel with five different pipelines implemented in MOTHUR and dada2. The obtained data sets (100%‐similarity OTUs and ASVs) were then compared against each other to investigate the quantity and quality of the conveyed information by (i) estimating sample‐wise sequence composition and diversity across methods, (ii) assessing tree‐inference difficulty, (iii) evaluating congruence of the derived phylogenies and (iv) correlating the obtained OTUs/ASVs with the existing genotaxonomic framework of 5S‐IGS types and lineages in *Fagus* (Denk et al. 2024). The obtained results will allow the optimisation of the 5S‐HTS workflow for future studies of phylogenetic/phylogeographic patterns in beech and other complex species systems, with emphasis on accuracy, easier manageability, interoperability and reusability of the produced data.

## 2 Material and Methods

### 2.1 Biological Material and DNA Sequencing

The genus *Fagus* L. (Fagaceae) comprises 14 tree species widely distributed in the temperate regions of the Northern Hemisphere and grouped into two main lineages: *Fagus* subgenus *Englerianae* (3 spp.) and *Fagus* subgenus *Fagus* (11 spp.; Denk et al. 2024). All extant species appear to be the product of dichotomous as well as reticulate speciation processes involving vicariance, genetic isolation (allopatric speciation) and hybrid (allopolyploid) origin, with clear evidence of allele/lineage sharing from both incomplete lineage sorting and recent interspecific gene flow (reviewed in Denk et al. 2024). 5S‐HTS data contain crucial information to investigate these multifaceted evolutionary processes; a clear‐cut reference backbone phylogenetic tree in which main 5S‐IGS lineages, sublineages and sequence types are detailed based on taxonomy, geography, molecular features and levels of ancestry was recently made available (Cardoni et al. 2022; Denk et al. 2024).

We selected six artificial (mock) samples with different levels of complexity (Table 1) from our earlier studies as test data set, each one representing a distinct species according to Denk et al. (2024). The seventh sample, representing the Taiwanese endemic species *Fagus hayatae*, was newly added in this study. Species were morphologically and genetically identified (Denk 1999a, 1999b; Shen 1992; Denk et al. 2024). DNA extractions were performed from silica‐gel dried leaves with the DNeasy plant minikit (Qiagen) and quantified with a NanoDrop spectrophotometer (TermoFisher Scientific). We pooled equal amounts of DNA from every individual up to a total of 20 ng per sample. Paired‐end Illumina sequencing (2 × 300 bp) was performed by LGC Genomics GmbH using the 5S‐IGS plant‐specific primer pair CGTGTTTGGGCGAGAGTAGT (forward) and CTGCGGAGTTCTGATGG (reverse). All primer‐clipped sequences are available in the Sequence Read Archive under BioProjects PRJNA681175 and PRJNA1019259.

**TABLE 1:** Investigated data set.

Sample no.	Species	Subgenus	Region	Site	Pooled individuals	References
04	*F. caspica*	*Fagus*	NC Iran	Noor	5	Cardoni et al. (2022)
05	*F. crenata*	*Fagus*	Japan	Various	5	Cardoni et al. (2022)
06	*F. japonica*	*Engleriana*	Japan	Various	5	Cardoni et al. (2022)
11	*F. orientalis* [s.str.]	*Fagus*	NE Greece	Dadia	5	Cardoni et al. (2022)
12	*F. sylvatica*	*Fagus*	C Germany	Göttingen	5	Cardoni et al. (2022)
25	*F. hohenackeriana*	*Fagus*	W Georgia	Ratscha	1	Denk et al. (2024)
26	*F. hayatae*	*Fagus*	NE Taiwan	Taipingshan	1	This study

As an accessory test, in order to check the wider applicability of our findings, we performed the same methodological comparison in three samples differing in taxonomy, geographic origin, ecological context and sample complexity: (i) a mock community sample of oaks (*Quercus*, Fagaceae; three distantly related species from a single North African population, originally studied with MOTHUR OTUs in Piredda et al. 2021); (ii) a yet unstudied single‐individual sample of European chestnut from Spain ( *Castanea sativa* Mill.; Fagaceae; 5S‐IGS so far unknown); (iii) a single‐species sample of *Viola arvensis* Murray (pooled DNA extracts from four different Italian populations, originally studied with MOTHUR‐OTUs in Scoppola et al. 2022).

### 2.2 Processing of Raw Data Using ASVs and OTUs‐Based Workflows

#### 2.2.1 MOTHUR OTUs‐Based Workflows

Illumina paired‐end reads (raw sequence data with adapters and primers clipped) were processed using MOTHUR v.1.41.1 (Schloss et al. 2009) using default parameters. Contigs between read pairs were assembled using the ΔQ parameter (Kozich et al. 2013) for solving base call differences in the overlapping region. No ambiguous bases were allowed, and sequences containing homopolymers longer than 20 nucleotides were removed. Reads were dereplicated and screened for chimeras using UCHIME in de novo mode (Edgar et al. 2011) producing the final dataset of OTUs with 100% sequence identity. MOTHUR produces OTUs, but an ASV generating option is also available. The method built into MOTHUR for identifying ASVs is the pre.cluster command that implements a pseudosingle linkage algorithm with the goal of removing sequences that are likely due to sequencing errors (Huse et al. 2010). The pre.cluster command was applied after the dereplication step and before the chimeras' screening, allowing for up to two nucleotide differences between sequences.

To filter potential artefacts and contaminating sequences in the comparison of the total number of sequences retrieved, the proportion of unique or shared sequences and sample diversity across the different pipelines, a cut‐off abundance ≥ 4 was applied to the total datasets obtained with both MOTHUR workflows (Bálint et al. 2016). A per‐sample cut‐off abundance ≥ 4 and, in addition, a cut‐off ≥ 25 on the MOTHUR data sets were applied in the phylogenetic analyses (see below).

#### 2.2.2 DADA2 ASV‐Based Workflows

Illumina paired‐end reads were trimmed based on quality score plots (forward: 200 bp, reverse: 150 bp); the filtered reads were then used to train the error model with the learnErrors() function. After the replication step, the inference of amplicon sequence variants (ASVs) (dada command) was performed applying the different options available (independent, nonpooled, samples: pool = FALSE; pseudopooled: pool = ‘pseudo’; and pooled samples: pool = TRUE) (https://benjjneb.github.io/dada2/pseudo.html#Pseudo‐pooling). By default, DADA2 processes each sample independently (‘no pool’ option). However, since pooling information across samples can increase sensitivity to sequence variants that may be present at very low frequencies in multiple samples, DADA2 offers the ‘pool’ option, with which all samples are pooled together for sample inference, and the ‘pseudopool’ option, where all samples are processed independently after sharing information, approximating pooled sample inference in linear time. Finally, the forward and reverse reads were merged and screened for chimeric sequences. All resulting unique ASVs were included in the downstream analyses.

At the end of the five workflows applied, the datasets were visually checked to remove unfiltered imperfect ends, and sequences longer than 340 base pairs (bp) and shorter than 200 bp were removed (see Cardoni et al. 2022, data S1). Codes for data processing and obtained multiple‐sequence alignments are included in the Supporting Information Archive (SDA, folders ‘1_Codes’ and ‘2_MSAs’; Simeone et al. 2024).

### 2.3 Comparison of Genetic Diversity and Phylogenetic Signals Provided by OTU/ASV Datasets Across Samples

The generated OTU/ASV data sets were analysed to assess diversity profiles (Shannon index; Shannon 1948) and private/shared representative sequences (Venn diagram) across the five workflows applied (MOTHUR‐OTUs and ‐ASVs, dada2 ‘pooled’, ‘pseudo‐pooled’, and ‘non‐pooled’ ASVs). Analyses were conducted in RStudio (RStudio Team 2020), with the packages ‘venn’ (Dusa 2022), ‘vegan’ (Oksanen et al. 2022), ‘readr’ (Wickham et al. 2024), and ‘tidyverse’ (Wickham et al. 2019).

To establish the relevance of the phylogenetic signals from the five workflows, fasta files generated for each of the seven samples were aligned using MAFFT v.7 (Katoh and Standley 2013). MAFFT‐generated multiple sequence alignments (MSAs) were manually checked and used for phylogenetic inference (trees and networks) individually or combined with the reference data set of Denk et al. (2024) to recognise and trace the clades representing the main types across samples and identify additional lineages or those not covered by the tip sets produced by each pipeline. In addition, we used Pythia (https://github.com/tschuelia/PyPythia), a lightweight python library, to predict the ruggedness of the MSAs and quantify tree‐inference difficulty (Haag et al. 2022). Maximum likelihood (ML) analyses relied on RAxML‐NG v.1.1.0 (Kozlov et al. 2019), and trees (SDA, folder 3_SampleWiseTrees) were inferred under a GTR + Γ substitution model; heuristic search for the best‐scoring ML tree topology and branch support were established via default parameters (20 tree searches using 10 random and 10 parsimony‐based starting trees; nonparametric bootstrap (BS) support using the default MRE‐based bootstopping test after every 50 replicates). Trees' visualisation was performed in iTOL (www.itol.embl.de; Letunic and Bork 2019) or DENDROSCOPE 3 (Huson and Scornavacca 2012). To summarise the differentiation capacity comprised by the OTU/ASV sets, that is, the intersample phylogenetic relationships, we inferred Neighbour‐Nets (NNet) based on ‘phylogenetic Bray‐Curtis’ distances (Göker and Grimm 2008) by treating the OTUs and ASVs of each sample as ‘associates’ and the sample itself as ‘host’ (association list included in SDA). The Neighbour‐Net algorithm implemented in SPLITSTREE 4 (Bryant and Moulton 2004; Huson and Bryant 2006) was used to generate planar (equal angle, parameters set to default) phylogenetic networks (SDA, folder 4_PBC Networks) and visualise general diversification patterns.

## 3 Results

### 3.1 Obtained Data Sets and Tip Size Reduction

Table 2 reports the results obtained with the five tested workflows. The total numbers of representative sequences showed strong differences between the two MOTHUR and the three DADA2 workflows, with the number of ASVs identified with DADA2 being smaller by a factor > 250 than when using MOTHUR (OTUs or ASVs). The largest part of MOTHUR OTUs and ASVs occurs at low abundances (< 4), contrary to DADA2 ASVs. After the delimitation of MOTHUR datasets based on cutoff abundance (*ab*) ≥ 4, the total number of MOTHUR ASVs was half the number of OTUs, but about three times the values generated with the DADA2 workflows. The three options within dada2, ‘pooled’, ‘pseudo‐pooled’ or ‘non‐pooled’, differ only marginally in the number of identified ASVs, with the highest numbers of ASVs identified with the ‘pooled’ option. Overall, the three DADA2 workflows resulted in a substantial reduction (> 80%) of the tip set compared with the original approach (Cardoni et al. 2022) using MOTHUR OTUs and an abundance threshold of ≥ 4. Considering MOTHUR OTUs and ASVs with abundance ≥ 25, the total numbers of representative sequences produced by the five workflows were very similar.

**TABLE 2:** Proportion of low‐frequent representative sequences for each method in the total data set.

	mothur		dada2 asvs		
	OTUs	ASVs	Pld	PsPld	NPld
Total number of representative sequences	240,001	247,423	854	904	901
With abundance ≥ 4	4860	2427	848	896	893
Proportion of repr. seq. with abundance < 4	98%	98%	0.7%	0.9%	0.9%
Total reduction factor^a^	1	0.499	0.176	0.186	0.185
Repr. seq. with abundance ≥ 25	807	718	703	715	712

Abbreviations: NPld, nonpooled; Pld, pooled; PsPld, pseudopooled.

^a^ Determined in relation to the original approach (Cardoni et al. 2022) using mothur‐defined OTUs and omitting the vast number of representative sequences with abundance < 4.

Table 3 details the numbers of the representative sequences obtained with the five pipelines in every sample, ranging between 62 and 222 total DADA2 ASVs, compared with 542–943 OTUs and 315–598 ASVs (*ab* ≥ 4) down to 76–178 OTUs and 61–167 ASVs (*ab* ≥ 25), respectively, produced by MOTHUR. Based on these numbers, DADA2 clearly outperforms MOTHUR (*ab* ≥ 4) in reducing the set of representative sequences, both in the total and sample‐wise datasets. Given the exiguous proportion of low abundance (*ab* < 4) ASVs detected with DADA2 (< 1% compared to ≥ 98%–99% when using MOTHUR, Table 2), and the overall low number of generated tips, there is also no reason to apply any further, artificial abundance threshold (e.g., *ab* ≥ 25, as in Piredda et al. 2021, guide‐tree in Cardoni et al. 2022; Tables 2, 3) to filter the obtained sequence sets for the subsequent phylogenetic analyses. Higher reduction factors can be achieved with MOTHUR data when using a high threshold (*ab* ≥ 25) but this comes at the price of losing rare but divergent and potentially interesting tips (see below). File S1 reports the working data sets used in the downstream analyses (MOTHUR OTUs/ASVs with abundance ≥ 4 and the total numbers of DADA2 ASVs).

**TABLE 3:** Tip set dimensions.

Sample/species	mothur, *ab*. ≥ 4			mothur, *ab*. ≥ 25			dada2 asvs			
	OTUs	ASVs	RF	OTUs	ASVs	RF	Pld	PsPld	NPld	RF
04—*F. caspica*	628	292	0.46	99	80	0.13–0.16	79	64	**62**	0.09–0.12
05— *F. crenata*	939	287	0.30	106	**88**	0.09–011	123	108	110	0.11–0.13
06— *F. japonica*	860	558	0.64	178	**167**	0.19–0.21	216	222	220	0.25–0.26
11— *F. orientalis*	561	277	0.56	111	**85**	0.15–0.2	118	94	92	0.16–0.21
12— *F. sylvatica*	943	500	0.49	177	**162**	0.17–0.19	179	172	172	0.18–0.19
25—*F. hohenackeriana*	542	280	0.52	101	**82**	0.15–0.19	132	127	130	0.23–0.24
26—*F. hayatae*	543	248	0.46	76	**61**	0.11–0.14	124	120	125	0.22–0.23

*Note:* Representative sequences produced by MOTHUR and DADA2 in each sample. ASVs determined by dada2 using three basic settings: NPld, nonpooled; Pld, pooled; PsPld, pseudopooled. Method producing highest reduction of tip set in bold font; RF, Reduction factor calculated as the number of representative reads (MOTHUR‐OTUs with *ab* ≥ 25, ASVs) divided by the number of MOTHUR‐OTUs (*ab* ≥ 4).

**FIGURE 1:**
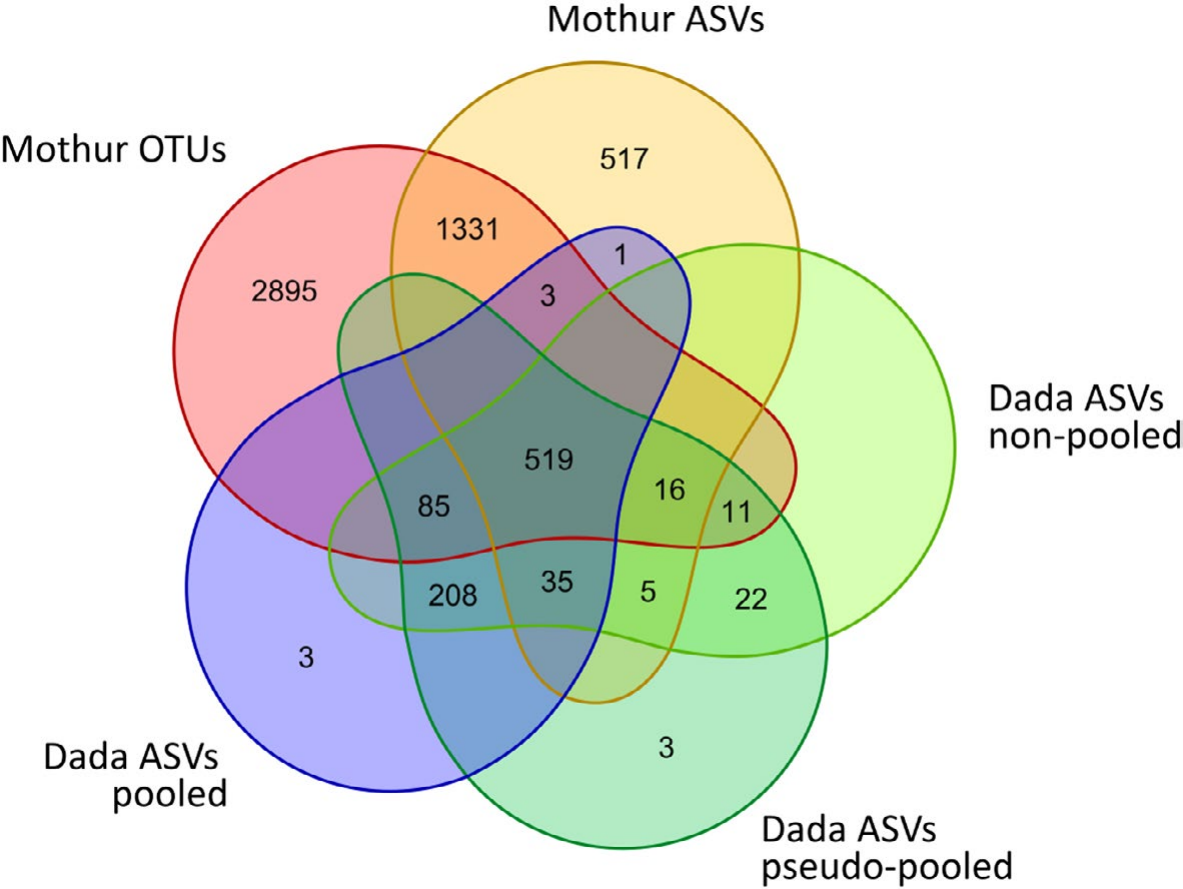
Venn diagram depicting the number of representative sequences unique to or shared across the different pipelines of data analysis.

The Venn diagram (Figure 1) correlating the ‘basic’ working datasets (all DADA2‐ASVs; MOTHUR‐generated OTUs and ASVs with *ab* ≥ 4) showed that 519 representative sequences (corresponding to 71.9% of reads) were shared across the five workflows. The largest part of private representative sequences was generated within MOTHUR (2895 OTUs and 517 ASVs), but they comprised only 2.6% and 1.2% of total reads, respectively. The two MOTHUR workflows shared 1869 representative sequences (79.2% of reads), whereas 4743 representatives (12.1% of total reads) were not found by any DADA2 working option; the three DADA2 workflows instead produced more overlapping data sets (911 shared representative sequences, corresponding to 87.9% total reads), with no or very few private representatives.

The sample diversity was calculated with the Shannon index (Table 4) that accounted for the number and the proportion of tips retrieved by ASVs and OTUs‐based workflows (i.e., the even/uneven distribution of reads in the generated representative sequence dataset). When using the cut‐off abundance ≥ 4, highest values were scored by OTUs in every sample; in contrast, MOTHUR ASVs scored lower values than DADA2 ASVs in two samples (#05, *F. crenata* , and #26, *F. hayatae*). Diversity values of the OTUs and ASVs produced by MOTHUR were only comparable within sample #04 (*F. caspica*), while no, or only negligible, variation among samples was found with the three different pooling strategies within DADA2. Interestingly, samples #06 ( *F. japonica* ) and #12 ( *F. sylvatica* ) showed the highest Shannon values across all workflows. On the other hand, slightly contrasting results were recorded in samples with low diversity scores: Sample #26 showed the lowest estimation with OTUs and nearly the lowest with all ASV methods, sample #05 showed the lowest estimation with MOTHUR ASVs and nearly the lowest with all other methods, whereas sample #04 showed the lowest Shannon values only with DADA2‐ASVs. The cut‐off abundance ≥ 25 applied to the MOTHUR data sets sensibly reduced the Shannon's estimates in all samples, still evidencing lowest diversity estimates in samples #26 and #05, but overlooking sample #04. The reason for this is the above‐mentioned cost for the tip‐size reduction of the MOTHUR *ab* ≥ 25 datasets: the pruning of rare but divergent variants found in the MOTHUR *ab* ≥ 4 data sets (retained in the DADA2 data sets) is lost in the MOTHUR *ab* ≥ 25 data sets. By comparison across MOTHUR and DADA2 sample‐wise trees, we identified 19 rare and divergent variants, including 12 potentially new types not covered or distinguished before (Table 5; File S2). Of these, eight OTUs and five ASVs are partially lost, together with eight OTUs and eleven ASVs totally lost in the MOTHUR *ab* ≥ 25 data sets versus 2–3/6 partially/totally lost in the DADA2 data sets (mostly types only detected in a single sample). One type, Lineage C variants, is generally not present in MOTHUR ASV‐trees with an abundance threshold of 25 and missing from all but one MOTHUR OTU‐tree.

**TABLE 4:** Diversity estimates (Shannon index) produced by the five methodologies (tip sets) in the investigated beech samples; [1] mothur OTUs; [2] mothur ASVs; A: abundance ≥ 4, B: abundance ≥ 25; [3] dada2 ASVs, pooled, [4]: pseudopooled, [5]: nonpooled.

Sample/species	04 *F. caspica*	05 *F. crenata*	06 *F. japonica*	11 *F. orientalis*	12 *F. sylvatica*	25 *F. hohenackeriana*	26 *F. hayatae*
**Tip set**							
[1A]	4.733	4.128	**5.159**	4.362	5.073	4.333	3.763
[1B]	3.808	2.820	**4.361**	3.489	4.272	3.475	2.937
[2A]	4.633	3.292	**4.893**	3.945	4.736	3.795	3.330
[2B]	3.949	3.237	**4.328**	3.596	4.289	3.605	3.007
[3]	3.338	3.347	**4.741**	3.678	4.472	3.651	3.397
[4]	3.337	3.350	**4.765**	3.700	4.480	3.680	3.421
[5]	3.330	3.352	**4.754**	3.694	4.480	3.680	3.424

*Note:* Colouring highlights highest (red) and lowest Shannon indices per sample (blue font); shading highlights the sample with the highest (bold font) and second‐highest Shannon index per methodology.

**TABLE 5:** Number of rare divergent 5S‐IGS sequence types identified by the most‐comprehensive MOTHUR *ab* ≥ 4 trees and the tip‐reduced MOTHUR *ab* ≥ 25 and DADA2 trees.

Sample, species	Tot	New	MOTHUR‐OTUs		MOTHUR‐ASVs		DADA2 ASVs		
			*ab ≥* 4	*ab* ≥ 25	*ab ≥* 4	*ab* ≥ 25	Npld	PsPld	Pld
04—*F. caspica*	9	4	All	4	5	2	7	7	8
05— *F. crenata*	5	1	All	None	All	None	All	All	4
06— *F. japonica*	1	1	None	None	None	None	None	None	1
11— *F. orientalis*	11	4	8	2	8	1	8^a^	8^a^	8^a^
12— *F. sylvatica*	10	5	All	2	All	None	8	8	6
25—*F. hohenackeriana*	8	3	All	All	4	4	7	7	7
26—*F. hayatae*	4	2	All	None	2	None	All	All	All

*Note:* Npld, nonpooled; PsPld, pseudopooled; Pld, pooled; Tot, total number of rare divergent types.

^a^ Of the 8 captured with the ‘pooled’ setting, only four were also captured with the other two settings of DADA2.

### 3.2 Effect of Tip Sample Size on Tree Inference and Topology

**FIGURE 2:**
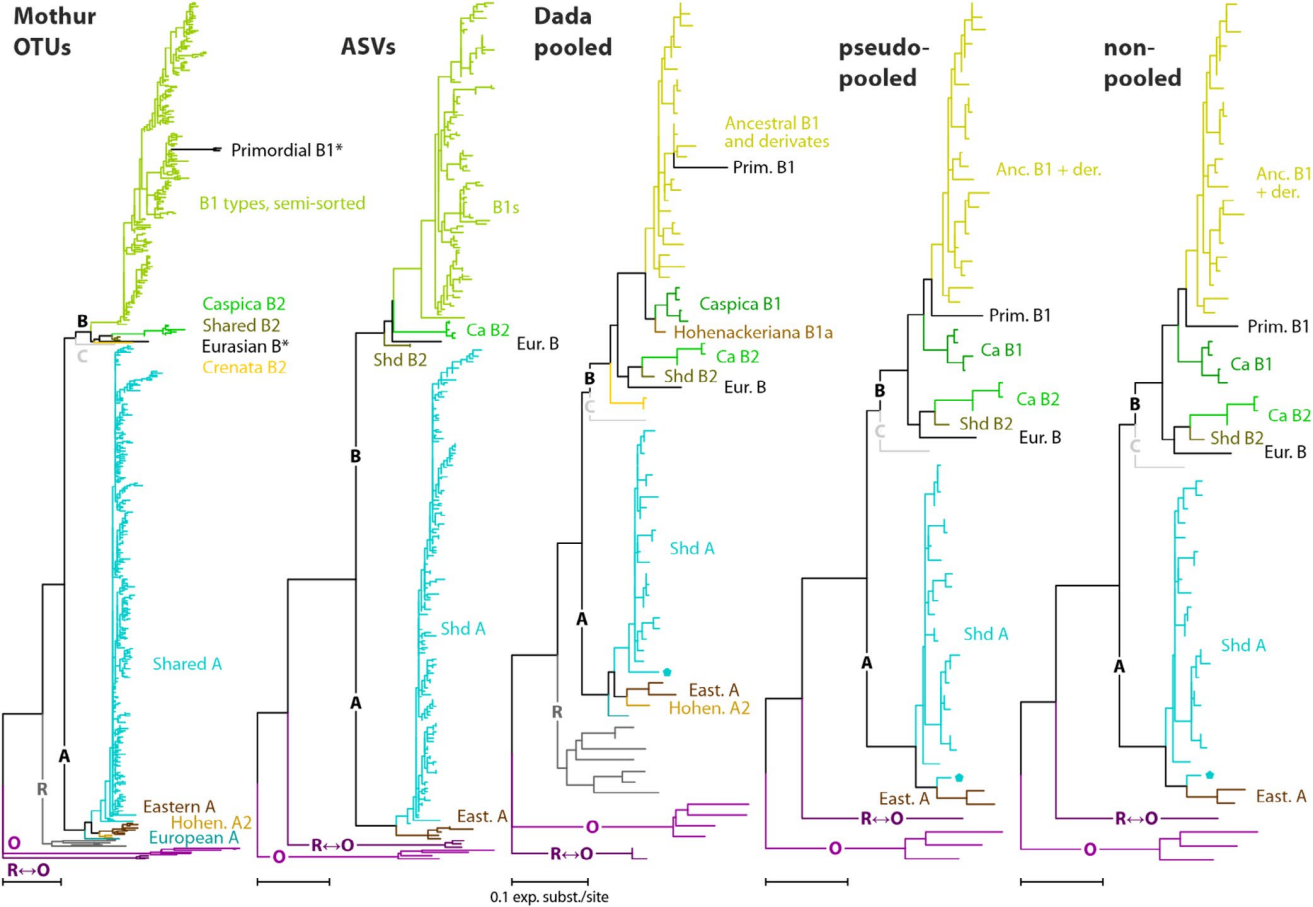
Full maximum likelihood trees for sample #04 (*F. caspica*). Main 5S‐IGS types are annotated, asterisks denote reference tips after Denk et al. (2024). Trees are ordered by increasing tip sample size reduction. ML trees of all other samples are provided as File S3.

**FIGURE 3:**
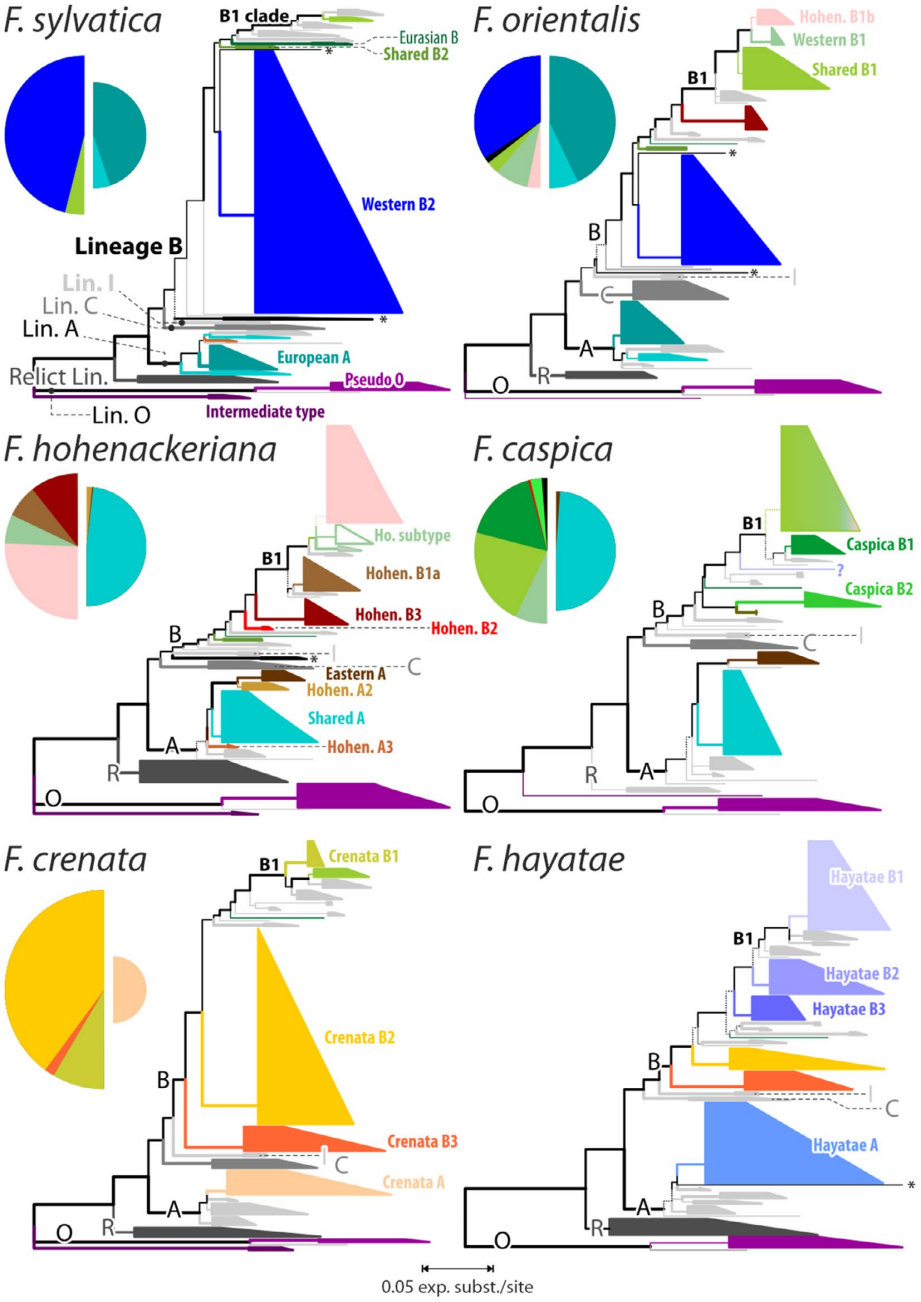
Collapsed maximum likelihood trees inferred on the DADA2 ‘non‐pooled’ ASV data sets and rooted on the Lineage O variants for the six samples belonging to *Fagus* subgen. *Fagus*, with the main types annotated (following Denk et al. 2024). For comparison, pie charts showing the relative abundance of main 5S‐IGS sequence variants as defined and discussed by Denk et al. (2024) are provided. *Fagus japonica* tree available in File S3.

The large majority of OTUs, and additional ASVs kept by MOTHUR, are sequences that are identical or very similar to each other and build up accordingly flat, comb‐like terminal subtrees (example given in Figure 2; for full trees and MSAs see SDA, which includes annotated per‐sample trees in nexml format). The branch support within these comb‐like subtrees was typically low (see for instance BS support of the ‘Shared A’ splits in File S2), indicating that most of the differences between the OTUs and ASVs of such terminal ‘combs’ are random and not sorted by evolutionary lineages (cf. Schliep et al. 2017). They are inference‐wise detrimental rather than being phylogenetically informative: Pythia‐predicted difficulty ranged between 0.54 and 0.62 for DADA2 ASV data across samples, while reaching 0.60–0.80 for MOTHUR OTUs and ASVs with *ab* ≥ 4 (File S2); tree inference difficulty remained higher (minimum difference range: 0.03–0.13) for MOTHUR OTU data and most ASV data (−0.02–0.16) using *ab* ≥ 25 thresholds relative to the DADA2 ASV data, albeit the latter produced a slightly higher number of tips. Among DADA2 workflows, ‘non‐pooled’ resulted in identical or slightly lower difficulty scores than ‘pooled’ and ‘pseudo‐pooled’. The much tip‐reduced DADA2 trees produced generally equal or slightly increased internal branch support (Table 6; File S2) and a topology well reflecting the reference tree for *Fagus* 5S‐IGS (File S3). Figure 3 shows that, despite much reduced, the dada2 ‘non‐pooled’ ASV tips cover all the expected main sequence variants (5S‐IGS main types as defined in Cardoni et al. 2022 and Denk et al. 2024). They also well reflect the overall diversity patterns within each main type subtree and the difference between the two main lineages of 5S‐IGS repeats, Lineage A and Lineage B.

**TABLE 6:** Bootstrap support for major splits (taxon bipartitions, clades in rooted trees) observed in 5S‐IGS maximum likelihood (ML) trees across samples and approaches.

Split, clade	06 *F. japonica*	26 *F. hayatae*	05 *F. crenata*	04 *F. caspica*	25 *F. hohenackeriana*	11 *F. orientalis*	12 *F. sylvatIca*
‘Ingroup’|‘outgroup’	100	100	≥ 90	93–96	93–99	≥ 96	≥ 92
Japonica O	26–36 [1B,2] Grade	—	—	—	—	—	—
Pseudo O	—	94–98^a^	90–98^a^	90–97^c^	≥ 96	≥ 98	89–99
Relict Lin.	—	65–66^a^ [2A] 88	80–89^a^	[1A] 86 [3A] 84 —^d^	90–93^e^ [1A] 79	[3] 96–98 [1,2] —^e^	89–99
A + B + C + I	+X: 43–44 [1B,2] n.r.	85–89 [1B] n.r. [2] 71,76	90–96 [2B] 63	94–96	91–94 [1B] 86 [2B] 84	88–90 [1B] 64 [2B] 74 [3A]: n.r.	92–98 [1B] 70 [2B] 63
Lin. A	—	96–98	≥ 97 [2B] —^b^	98–99	≥ 98	[1A,2A] 68 [1B] 87 [2B] 90 [3] 98–99	93–99
B + I	16−44 [2B] n.r.	71–78 [1B] 64 [2B] 67	67–75	61–68	47–57 [1B] 76 [2B] 72	36_[3A]_–72_[1B]_	56–58 [1A] 41 [1B] 75 [2B] 76
Lin. I	50–80	—	—	—	—	—	—
Lin. B	—	61–72 [1B] 57	61–71	53–58 [1A] 73	56_[3C]_–72_[1B]_	13_[3A]_–63_[1B]_	33–43 [1B] 65 [2B] 72

*Note:* [1] mothur OTUs (A: *Ab* ≥ 4, B: *Ab* ≥ 25); [2] mothur ASVs (A: *Ab* ≥ 4, B: *Ab* ≥ 25); [3] dada2 ASVs (A: pooled, B: pseudopooled, C: nonpooled). n.r., not realised in optimised ML tree. Japonica O and Lineage I variants are only found in *F. japonica* ; Pseudo O, Relict Lineage, Lineage A and Lineage B are only found in species of *F*. subgenus *Fagus*.

^a^ Only reference data Pseudo O and Relict Lineage variants present in MOTHUR *ab* ≥ 25 trees.

^b^ No Lineage A MOTHUR‐ASVs sampled with *ab* ≥ 25.

^c^ No Pseudo O MOTHUR‐ASVs sampled with *ab* ≥ 25.

^d^ No Relict Lineage representative sequences sampled in the other approaches.

^e^ No Relict Lineage MOTHUR‐ASVs sampled with *ab* ≥ 25.

With respect to the tree topologies inferred for the seven test samples, the much higher reduction factor of the DADA2 workflows is not prone to lose important variants and subtypes (Table 5; File S3). In contrast to the MOTHUR *ab* ≥ 25, the DADA2 reduced tip sets are a good reflection of the known signal in the quasi‐total set of representative reads. In addition, the OTUs and ASVs not recognised by DADA2 can have a detrimental effect on the inferred trees and underlying alignments: some clades representing types or subtypes with consistent placement in the tip‐reduced trees (DADA2, MOTHUR *ab* ≥ 25) may be differently placed in the MOTHUR *ab* ≥ 4 trees (File S2).

Among the rare, divergent variants (File S2) identified in the DADA2 trees are sequence variants that indicate a stronger connection between the East Asian species and some of the western Eurasian species. Based on the DADA2 trees, we inferred four new potentially diagnostic main types of sequence variants in the newly studied *F. hayatae* sample #26 (Hayatae A, Hayatae B1–B3; *N. Taiwan* ) in addition to ASVs shared with the other East Asian species sample #05, representing a mock population of *F. crenata* sampled across the Japanese archipelago (Crenata B2 and Crenata B3; File S3). Based on their placement in the DADA2 trees, Hayatae B1 constitutes rare variants in sample #05 and Hayatae B2 in sample #04, *F. caspica* (easternmost of W. Eurasian spp.). Variants classified earlier as Primordial B1 in sample #04 may represent Hayatae B1 variants as well, or pseudogenic counterparts. Pseudo O variants detected in all Western Eurasian species are found in sample #26 as well. One rare, divergent, and early diverged variant/type is limited to the western samples (#11, #12, and #25; *F. orientalis, F. sylvatica*, and *F. hohenackeriana*); another with affinity to Western B2, a type specific for the westernmost species pair *F. sylvatica‐orientalis* exclusive to samples #11 and #12, representing this species pair (Table 6).

### 3.3 Capturing Major Evolutionary Signals Across Approaches

**FIGURE 4:**
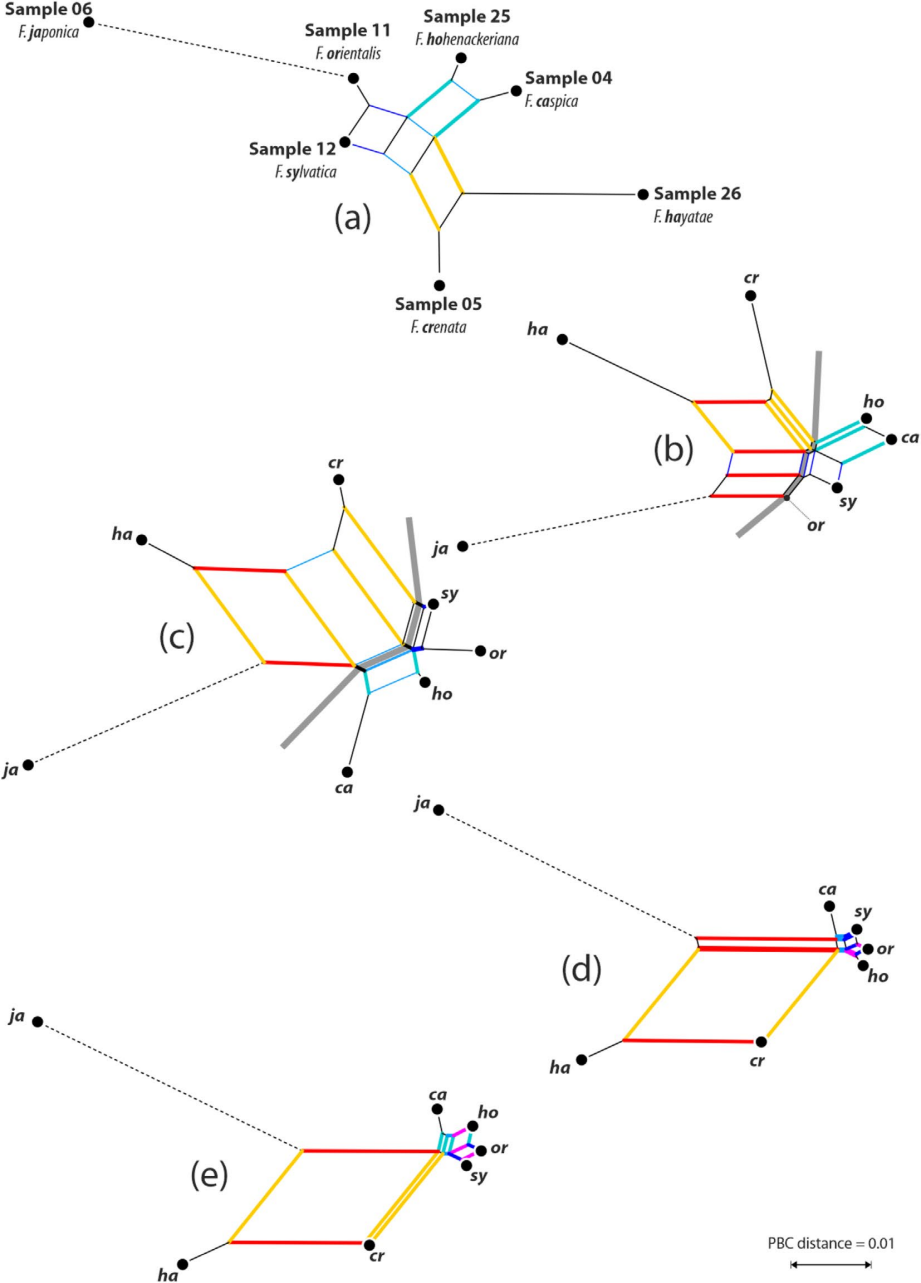
Neighbour‐nets based on intersample ‘phylogenetic Bray–Curtis’ distances, using as ‘associates’: (a, b) MOTHUR‐OTUs/‐ASVs with an abundance of ≥ 4 (a: OTUs; b: ASVs); (c–e) DADA2 ASVs (c: ‘Pooled’; d: ‘Pseudo‐pooled’; e: ‘Nonpooled’ options). Edge bundles of corresponding and expected neighbourhoods coloured accordingly, thick lines indicate neighbourhoods in line with known phylogenetic relationships. Grey line (b, c) refers to the edge bundle reflecting a split into Western Eurasian and East Asian species. All graphs have the same scale, terminal edge leading to sample #06, *F. japonica* (stippled), has been reduced by a factor of 2.

**FIGURE 5:**
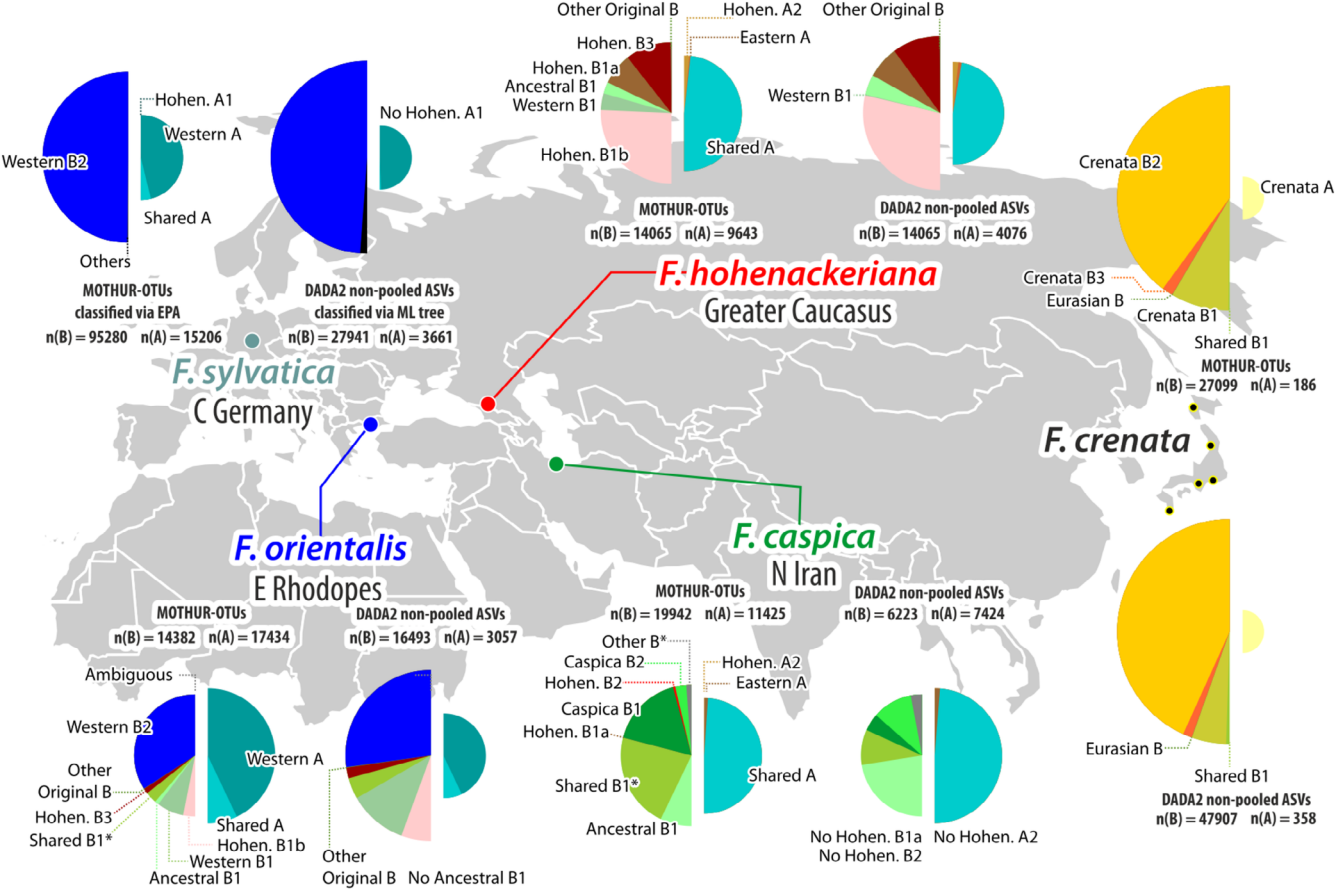
Map of the investigated beech samples with pie charts showing the relative abundance of main 5S‐IGS sequence variants; phylogenetic classification of MOTHUR‐OTUs (*ab* ≥ 4) and nonpooled ASVs based on the sample‐wise trees and EPA (cf. Cardoni et al. 2022).

The PBC networks (Figure 4) show the evolutionary signals captured by the five workflows (MOTHUR‐OTUs, ‐ASVs, *ab* ≥ 4; DADA2‐ASVs, pooled, nonpooled, pseudopooled) and their potential to infer evolution and differentiation processes in beech. All networks are in general agreement with the evolutionary history of *Fagus* as sketched in Cardoni et al. (2022) and refined by Denk et al. (2024). Beech 5S‐IGS lineages and main types are detailed in File S3. The ‘outgroup’ tip comprising mainly Lineage O and I 5S‐IGS variants (only found in *F. japonica, F*. subg. *Englerianae* but not in species of *F*. subg. *Fagus*) and a few Relict Lineage and Lineage X variants (sample #06, *F. japonica*, the only species of *F*. subg. *Englerianae* included in the dataset; cf. File S3), is most distant from all other tips (‘ingroup’, 5S‐IGS variants unique to members of *F*. subg. *Fagus*), including samples mostly composed of Lineage A and B variants, with a few tips representing pseudogene variants of Lineage O, Relict Lineage variants and Lineage C variants. The two tips representing the East Asian species, samples #05 (Japanese *F. crenata* ) and #26 (Taiwanese *F. hayatae*), are less distant and are part of the same neighbourhood with a more (DADA2‐ASV data, *ab* ≥ 4, Figure 4c–e) or less pronounced edge bundle (MOTHUR‐ASV data, *ab* ≥ 4, Figure 4b). This graph aspect, missing from the OTUs network (Figure 4a), reflects their partially species‐specific and partially shared Lineage B types composed of 5S‐IGS variants that are markedly and consistently distinct from the Lineage B variants of the Western Eurasian species samples #04 (*F. caspica*), #11 ( *F. orientalis* ), #12 ( *F. sylvatica* ) and #26 (*F. hohenackeriana*). In all ASV‐based graphs, a prominent box is formed by the *crenata‐hayatae* and a *hayatae‐japonica* neighbourhood. This is because there are no Lineage A and Lineage B 5S‐IGS types shared between *F. hayatae* and the Western Eurasian species. In contrast, there are a few Lineage A and B 5S‐IGS types shared between *F. crenata* and the Western Eurasian species, and one specific *F. crenata* type is very similar to a group of Lineage B variants shared by two or more Western Eurasian species. With respect to the East Asian species, the Western Eurasian species are notably similar, and, to various degrees, connected by small edge bundles forming neighbourhoods that are in line with their geographic provenances; notably, the DADA2 non‐pooled network captures all expected neighbourhoods (Table 7). The much‐decreased edge lengths in the DADA2 PBC graphs reflect the high genetic coherence of the Western Eurasian species, all sharing a common, exclusive ancestor: (i) the similarity in the population of found 5S‐IGS types (the kind of ASVs determined for each sample), and (ii) the amount of sequentially similar variants (ASVs). In the MOTHUR data sets, the West Eurasian samples differ more strongly in their OTU/ASV population. This difference is also reflected in the geographical patterns of total type abundances mapped in Figure 5.

**TABLE 7:** Observed and expected neighbourhoods in the PBC networks across workflows.

Neighbourhood	MOTHUR, *ab* ≥ 4		DADA2 ASVs		
	OTUs	ASVs	Pooled	Pseudopooled	Nonpooled
*Hayatae‐japonica*	No	Yes	Yes	Yes	Yes
*Crenata‐hayatae*	No	Yes	Yes	Yes	Yes
*Sylvatica‐orientalis*	No	No	No	Yes	Yes
*Orientalis‐hohenackeriana*	No	No	No	Yes	Yes
*Hohenackeriana‐caspica*	Yes	Yes	Yes	No	Yes

Our findings on the accessory tests performed on other plant genera with different evolutionary dynamics or eco‐geographical contexts are shown in File S4. In the oak mock sample, all methods unambiguously sorted all 5S‐IGS variants into distinct 5S‐IGS phylogenetic clades that were consistently assigned by BLAST to the target and/or sibling species; despite the much‐reduced ASV data set relative to the MOTHUR‐generated datasets with abundance cut‐off ≥ 4, DADA2 effectively recognised the presence of the species occurring in the lowest amount. Likewise, the topology of the trees inferred in the chestnut sample was congruent across methods. The 5S cistron of this species showed an extremely high number of low‐divergent units, strongly complicating all downstream analyses. Primary informative phylogenies were thus produced with MOTHUR‐generated data sets with abundance cut‐off ≥ 25. Three major clades were produced, characterised by a very low overall divergence and bootstrap support, that were resolved into six basic haplotype‐like groups; DADA2 demonstrated a better ability to manage the high stochasticity of most point mutations and detected all haplotype groups more clearly than MOTHUR (*ab* ≥ 25). Finally, the three methods displayed high congruency of results also in the *V. arvensis* sample, evidencing broad OTUs and ASVs co‐occurrence in all major clusters and subclusters produced in the Neighbour‐Net. In this case as well, the obtained MOTHUR data sets had to be reduced with an abundance cutoff > 25, given the high numbers of sequences retrieved and the intragenomic complexity of the organism at *ab* ≥ 4. Interestingly, DADA2‐ASVs further recognised an additional subcluster, unresolved by MOTHUR. In all samples, the reduction factor obtained with DADA2 (up to 0.10) was determinant for easier data exploration and analysis.

## 4 Discussion

Amplicon sequencing is among the most widely used High‐Throughput Sequencing (HTS) methods for numerous research and practical applications, including genome evolution, taxonomic and evolutionary analysis, community composition and functioning (Liu et al. 2020). Our previous HTS studies described the composition of the nuclear ribosomal 5S arrays in *Fagus*, showing the intragenomic coexistence of two loci with allopolyploid origin, together with lineage‐specific, relict, and unit types shared across species and biogeographic regions (Cardoni et al. 2022; Denk et al. 2024). However, because of the large amount of data (MOTHUR‐derived OTUs with a 100% identity threshold) retrieved in each sample (pooled DNA extracts of 1–5 individuals) and the computational/statistical effort required to extend our investigations to multiple individuals, populations and species, we explored alternative pipelines making downstream analyses easier and more efficient. Here, we demonstrate that DADA2‐derived amplicon sequence variants (ASVs) can efficiently replace MOTHUR‐derived operational taxonomic units (OTUs) in amplicon data preprocessing of a marker with high phylogenetic content.

### 4.1 Representativeness of OTU/ASV Data sets for Assessing Quasi‐Quantitative Biodiversity and Potential Sources of Error and Convenience of the Two Tested Pipelines

Picking up representative sequences as informative proxies for species identity, evolutionary dynamics, and ecological diversity is a key step in organismal metabarcoding as well as phylogeographic studies using HTS.

In environmental studies, a threshold of 97% sequence identity of the targeted 16S rRNA, 18S rRNA, or ITS gene regions is generally used to define OTUs at the species level. However, this threshold has recently been challenged, as clustering percentages should probably be adjusted depending on data complexity and the study objective (Cholet et al. 2022). On the one hand, clustering at low similarity percentages (e.g., 95%–97%) may fail to detect minor, yet relevant, differences among sequences (e.g., rare strains and species, key molecular variants). On the other hand, strict thresholds (e.g., 99%–100%) may inflate a dataset with some proportions of redundant sequences and artefacts (generally resulting in phylogenetically inconsistent OTUs; Brown et al. 2015; Chiarello et al. 2022) difficult to recognise, especially when phylogenetically high divergent groups are expected in the investigated community (Koeppel and Wu 2013). For this reason, abundance cutoffs (removal of low abundance OTUs) are generally recommended (Bálint et al. 2016), although there is no consensus on the optimal limit of sequence numbers that should be kept without affecting the completeness of the OTUs dataset (Cholet et al. 2022). For the present application, while necessary to reduce the number of OTUs, the choice between sequence identity or abundance thresholds is not straightforward from a principal‐theoretical point of view (discussed in Piredda et al. 2021; Cardoni et al. 2022): each OTU may or may not accurately represent a genuine repeat in one of the thousands of copies per array.

In recent times, the denoising approach generating ASVs has been promoted as a possible solution to solve these issues and, ultimately, to replace OTUs (Callahan et al. 2016). This approach is particularly attractive as it can resolve ‘actual’ (and not ‘consensus’) sequences differing by as little as a single nucleotide; denoising is performed by an algorithm so that no user‐defined abundance cutoffs are needed, thus increasing the results' reliability. In addition, different ASV data sets can be directly compared, facilitating comparison of data among different studies and implementation with increasing sampling designs (Callahan et al. 2017). These benefits may apply to our data and further studies focused on understanding genetic diversity and evolutionary dynamics of species and communities as well.

Several studies have compared the ASV method to the OTU approach using the classical ribosomal markers (16S rDNA, 18S rDNA and the ITS region) in mock communities. Overall results indicate that better precision (i.e., all sequences/taxa are detected with no false positives) and composition (i.e., relative abundance of the detected sequences) can be generally obtained by the former method (Bakker 2018; Pauvert et al. 2019; Moossavi et al. 2020; Prodan et al. 2020; Cholet et al. 2022). Conversely, OTUs generally achieve higher richness (i.e., number of sequences obtained) and stronger sensitivity (i.e., detection of rare sequences/taxa) (Prodan et al. 2020; Joos et al. 2020). Nevertheless, the impact of the two methodologies on large‐scale ecological patterns still needs to be fully elucidated (Straub et al. 2020; Chiarello et al. 2022), and some researchers suggest that the biological conclusions derived using either method can be largely consistent (e.g., Cholet et al. 2022).

In the present study, the efficiency of MOTHUR‐ (using two commonly applied abundance thresholds, *ab* ≥ 4 and *ab* ≥ 25) and DADA2‐derived OTUs and ASVs in respect to richness, precision, sensitivity and composition was evaluated against the background of the recently proposed framework of *Fagus* evolution and diversity (Denk et al. 2024), and in terms of statistical support in tree inference, ease of visualisation and computational effort. As expected, absolute richness, as expressed by the number of representative sequences retrieved (Tables 2, 3), was notably higher for the OTUs than the ASV data sets, and the occurrence of rare sequences was the most relevant difference among the tested workflows. Concerning precision, OTUs and ASVs produced mostly consistent results with regard to the captured phylogenetic signals (inferred trees, detected main 5S‐IGS types), and the subsequent relationships and relative differences between samples (Tables 4, 5, 6; Figures 2, 3; Files S2, S3). However, inferences using OTUs (*ab* ≥ 4) appear more prone to reconstruction artefacts (cf. PBC networks in Figure 4): pairwise distances and according edge lengths in the PBC graphs are inflated because of the sequence variation in, and the number of (cf. Göker and Grimm 2008), distinguished MOTHUR‐derived OTUs (and ASVs, to a lesser degree).

The strong reduction (up to ca. 90% for DADA2 and MOTHUR OTUs and ASVs with abundance ≥ 25) of the 5S‐IGS sequence variants obtained (Tables 2, 3) resulted in easier‐to‐handle but equally comprehensive data sets (see also File S4 for additional nonbeech samples), indicating that sensitivity regarding phylogenetic inferences was equally effective across workflows. The strong reduction is predominantly due to the large number of rare/unique sequence reads assembled into OTUs and ASVs by MOTHUR, whereas a large part (> 70%) of the processed reads with abundance > 4 coincides across all methods (Figure 1). Even though ASVs cannot be considered equivalent to 100%‐similarity OTUs (Edgar 2017, 2018; Callahan et al. 2017), this finding indicates that, at least in *Fagus*, OTUs retrieved beyond a certain abundance cut‐off (e.g., *ab* ≥ 25) match the denoised ASVs in number but not representativeness (Table 3). The same was observed for the additional nonbeech samples (File S4). An abundance threshold of ≥ 25 was used in our previous phylogenetic analyses (Piredda et al. 2021; Cardoni et al. 2022) based on preliminary correlations of specific sequence features (GC content, sequence length, molecular evidence of starting pseudogeny) and abundance. However, the high number of low‐abundance OTUs (< 25) should not be considered sequencing artefacts, as their estimated evolutionary signals (measured with the Evolutionary Placement Algorithm; Berger et al. 2011; Piredda et al. 2021) did not produce distortion in the inferred phylogenetic relationship but support the emergent differentiation patterns (Denk et al. 2024). Especially when studying new genera (chestnut sample in File S4) and fishing for rare, shared types (genetic relicts), the strong tip‐size reduction achieved with the DADA2 pipeline is advantageous because no abundance threshold to further filter the otherwise hard‐to‐handle number of representative sequences is needed.

Finally, we tested the application of the Shannon index as a proxy of the complexity of composition in the investigated samples. Notably, OTUs and ASVs identified the highest diversity in the same samples (#06, *F. japonica* , and #12, *F. sylvatica* ), whereas, concerning the identification of samples with lowest diversity, DADA2 ASVs seemed to combine the partially contrasting results retrieved with MOTHUR OTUs and ASVs with both *ab* ≥ *4* and *ab ≥ 25* (samples #05, *F. crenata* , and #26, *F. hayatae*), and identified a third further sample (#04, *F. caspica*). However, diversity should better be compared across communities that have been sampled equivalently, and our mock samples indeed represent different levels of complexity (see Table 1). Hence, strictly applied, all workflows indicate that, across a common range, *F. japonica* has more 5S‐IGS diversity than *F. crenata* , while the German population of *F. sylvatica* is more diverse than the Greek population of its sister species *F. orientalis* , and a single tree of the relatively widespread Caucasian *F. hohenackeriana* can be as diverse as populations comprising five individuals, in contrast to the individual of Taiwanese endemic *F. hayatae*. All these findings suggest complex evolutionary events enhancing or suppressing the molecular complexity of species, populations and individuals, and indeed require more extensive metapopulation, multispecific studies to be adequately assessed. The only inconsistency between MOTHUR and DADA2 workflows refers to Iranian *F. caspica*, resulting as the least diverse with DADA2 ASV data sets, as could be expected for a narrow‐endemic relict species (cf. Denk et al. 2024), in contrast with all MOTHUR‐generated data. Figure 2 and File S3 show higher amounts especially of ‘Shared A’ representative sequences detected by MOTHUR (*ab* ≥ 4), compared to DADA2. However, as documented in Denk et al. (2024), ‘Shared A’ sequences are unspecific variants in *F. caspica* (in sequence features and amounts), the most relevant being the ‘B types’ (both specific and primordial variants; all adequately detected by DADA2, see Figures 2, 3 and File S3). Thus, only unspecific sequence variants with no unique phylogeographic signal inflate the Shannon index results of MOTHUR data sets, masking the true population status (low diversity) contrary to DADA2. Notwithstanding the potential importance of rare sequences, in a genetic diversity study angle, lower estimates can imply isolation, drifting and inbreeding of individuals and populations; a correct estimation of the residing diversity, limiting the impact of rare, low‐informative sequences, can be advantageous for a clear identification of these vulnerable subjects and to inform conservation plans.

Therefore, the obtained DADA2 ASV datasets showed to be a good trade‐off compared to MOTHUR derived OTUs and ASVs for all studied samples (beech test set, additional samples covering species from other genera, geographical and ecological contexts), in terms of data richness, precision, sensitivity, composition and consistency of results. The reliability of DADA2 workflows, coupled with their interchangeability across studies and laboratories, and the potential to expand over time, appear promising also in relation to Shannon's and (possibly) other genetic diversity estimates (Straub et al. 2020; Chiarello et al. 2022). Indeed, their use in exhaustive metapopulation studies could be useful to complement current studies on forest biodiversity, including the identification of geographic structures of gene diversity, diverging/drifting in marginal or isolated populations, hybridisation in contact zones and relevant genepools for conservation and/or germplasm banking.

### 4.2 Representativeness of OTU/ASV Collections for Phylogenetic and Taxonomic Questions

Intragenomic variation of the 5S nrDNA arrays has been observed in several organisms, including plants (Wang et al. 2023). Primary causes for intragenomic 5S nrDNA variation are whole genome duplication (autopolyploidisation), incomplete concerted evolution, hybridisation (including allopolyploidisation) and introgression. Secondary causes involve intragenomic recombination between distinct maternal and paternal arrays (homoeologous arrays in the case of allopolyploids), interarray competition culminating in the silencing of one parental array leading to pseudogeny and intracellular DNA and RNA repair mechanisms (Hemleben et al. 2021 and literature cited therein). Additionally, some (especially the deleterious) mutations can be reversed by new copies, consistent with the birth‐and‐death model of multigene family evolution (Nei and Rooney 2005). In a tree genus like beech, additional bioecological factors such as their longevity, temporally small population size (e.g., during Pleistocene cold phases) associated with increased genetic drift, and wind pollination may facilitate incomplete homogenisation within and between populations. The results are genetic mosaics, with a high level of heterozygosity across nuclear gene regions (Jiang et al. 2021; Li et al. 2023) and extreme levels of nrDNA spacer diversity (Denk et al. 2002, 2024; Cardoni et al. 2022). High‐throughput sequencing (HTS) is a powerful tool to investigate the multiple facets of such a complex model of evolution by combining the benefits of cloning to capture intra‐genomic (here: intra‐sample) nrDNA variation with the speediness and data amount introduced by next‐generation sequencing approaches. On the other hand, random noise as well as method‐induced artefacts (sequencing errors, contamination) can be detrimental for the reconstruction of interpopulation and interspecific relationships.

We recorded only minor topological differences between methods and approaches. Most importantly, the clades representing main sequence types as defined by Cardoni et al. (2022) and Denk et al. (2024) have about the same branch support, irrespective of the used pipeline and its resultant number of tips (OTUs or ASVs; Table 6; File S2), indicating that all methods and settings produce a set of OTUs and ASVs that are equally representative for each sample (cf. Cardoni et al. 2022; Denk et al. 2024). The much higher number of MOTHUR‐generated OTUs and ASVs reflects additional intratype variation (terminal noise). Terminal noise, expressed by flat terminal subtrees, is unfavourable for tree inference because the optimisation needs to gauge between topological alternatives with similar likelihoods and ambiguous branch support differing only by permutations of how to place highly similar to near‐identical tips. As such, they will not only increase computation time but also the difficulty (already high, Pythia scores > 0.5; File S2) of inferring phylogenetic trees (Stamatakis 2015; Haag et al. 2022). By reducing the number of representative tips by a factor of four to ten using DADA2 or a cut‐off abundance ≥ 25 for MOTHUR (Table 3 but see Table 5), phylogenetic reconstructions can be done much more efficiently. If needed, for example, in case of tips with unreasonable long branches or unexpectedly placed tips, the underlying alignments can be more easily explored as well.

Based on the per‐sample phylogenetic trees, the much fewer DADA2‐generated ASVs outperform the much larger MOTHUR (*ab* ≥ 4) tip sets not only in their handling during tree inference or post‐analysis; they are also not missing any relevant, for taxonomic and phylogenetic questions, 5S‐IGS lineage/group of variants (Figure 4; Files S3, S4). In combination with ‘phylogenetic Bray‐Curtis’ distances (Göker and Grimm 2008), they also better reflect the genetic distances between samples (here: populations and species). Thus, the ASVs derived from the DADA2 pipeline allow for efficiently reducing redundancy while producing the number of necessary tips for phylogenetic (Figure 4; Table 6) and principal taxonomic assessments (e.g., when applying the genotaxonomic multifurcating key introduced by Denk et al. 2024). Comparing the here tested model samples with the 42‐tip reference tree and matrix (Denk et al. 2024), the best fit is found with the ‘non‐pooled’ option of DADA2, being, in addition to ‘pseudo‐pooled’, the approach resulting in the smallest possible set of representative sequences (Table 3).

It is also worth noting the perfect agreement between the Shannon diversity estimates and the phylogenetic reconstructions obtained with DADA2 ASVs. The two Japanese samples (#05 and #06, multiple individuals of *F. japonica* and *F. crenata* representing different populations) score quite different diversity values, as do the two European species (#11 and #12, multiple individuals of *F. sylvatica* and *F. orientalis* from single populations), and samples #25 and #26, comprising single DNA extracts of *F. hohenackeriana* and *F. hayatae*. This concurs with what we see in the per‐sample trees (Figure 2; File S2). Both main and codominant lineages characterising the 5S‐IGS gene pool of *F. japonica* , Lineage I and especially Lineage O, are relatively diverse (forming well developed, large subtrees with ± long internal branches), especially when compared to the A‐lineage types (co‐)dominant in most Western Eurasian species (see also Cardoni et al. 2022; Denk et al. 2024) and in *F. crenata* (sym‐ to parapatric with *F. japonica* ). The relatively high Shannon index for the *F. sylvatica* relates to the diversity within its dominant Lineage B variants, the Western B2 type (cf. Cardoni et al. 2022).

Thus, we recommend using the nonpooled ASVs option for the primary diversity assessment and phylogenetic analyses for future samples and populations of beech species, in particular when studying new species. For instance, the Taiwanese *F. hayatae* sample is characterised by four new, potentially specific A‐ and B‐Lineage main types (Figure 3; Hayatae A, B1–B3), while sharing the Crenata B2 and B3 types but missing the Crenata A (a distant relative of Hayatae A within Lineage A) and Crenata B1 type (a sibling type of Hayatae B1). A data situation well reflected in the according DADA2‐based PBC distances and subsequent networks (Figure 4c–d, in contrast to Figure 4a,b). Same benefits are expectable in other plant genera (File S4). The methodological comparison performed across additional samples consisting of multiple or single individuals of one or more tree or plant species, including allopolyploid and largely hybridising species from different ecological and geographic contexts, reinforced the clear evidence of the complete and consistent phylogenetic reconstructions, coupled with easier data handling, reliability and reusability, that can be obtained with the much‐decreased DADA2‐ASV data sets.

On these grounds and given the general complexity of such data and interpretations, we propose that the DADA2 pipeline may outperform the MOTHUR pipeline(s) for similar studies in other organisms with complex bioecological and evolutionary dynamics. Nevertheless, MOTHUR OTUs can still be of importance in benchmark studies on newly investigated species: for a practical evaluation of the genome complexity where rare sequences/lineages with crucial biological roles must not be overlooked, or where the aim is to assess and map, for example, using the Evolutionary Placement Algorithm (EPA), absolute diversity. A typical experimental layout for a genotaxonomic study of a new plant genus or group of plant species could be to first process the raw HTS data using DADA2 (Step 1); infer a comprehensive backbone tree using the DADA2‐obtained ASVs to identify main lineages and variants (clades, sequence types; Step 2); select representative ASVs for a reference matrix and phylogenetic tree (Step 3); and finally classify MOTHUR‐obtained OTUs (100% identity, abundance cut‐off ≥ 4) using EPA and the DADA2‐derived reference tree and matrix.

## 5 Conclusion

High‐throughput sequencing of highly divergent, multicopy nuclear gene regions such as the nontranscribed intergenic spacers of the 5S nrDNA arrays represents a new and promising tool to investigate evolutionary histories and genetic resources of plant species, provided the data can be processed efficiently. This work compared the efficacy and efficiency of DADA2‐derived amplicon sequence variants (ASVs) and MOTHUR‐derived operational taxonomic units (OTUs) in amplicon data preprocessing of the 5S‐IGS in *Fagus*.

We showed that (i) differences in the number of sequences recovered are predominantly due to rare operational units, which are more prevalent in the OTU‐based clustering approach; (ii) the phylogenetic conclusions that can be drawn using either method are largely consistent, thus indicating that differences in sequence variation detected by the two approaches are minimal and of no phylogenetic relevance; (iii) the DADA2‐based ASV datasets were easier to handle and without the potentially detrimental effects of redundancy and noise conveyed by MOTHUR OTUs. Therefore, the reduction of representative sequences (i.e., ‘pruning’ the structurally unnecessary tips in a phylogenetic tree) provided by DADA2 ASVs has important benefits: complete and consistent phylogenetic reconstructions can be obtained; data handling can be performed more easily, data are reusable across studies and can be implemented in future data sets. DADA2 ASVs may thus efficiently replace OTUs in future studies aimed at deciphering complex bioecological phenomena such as reticulation, polyploidisation, hybridisation, drift and inferring evolutionary pathways in complex species systems, especially when using highly variable markers and increasing sample sets. However, if the objective is to draw absolute (or total) levels of diversity and quasi‐proportional composition of gene pools at and below the species level, MOTHUR‐OTUs datasets (100% identity, *ab* ≥ 4) may be more comprehensive, thus worthy of parallel inspection.

## Author Contributions

**Simone Cardoni:** data curation (equal), formal analysis (equal), investigation (lead), writing – review and editing (equal). **Roberta Piredda:** conceptualisation (equal), data curation (equal), methodology (lead), writing – review and editing (equal). **Guido W. Grimm:** conceptualisation (equal), formal analysis (equal), validation (lead), visualisation (lead), writing – original draft (equal). **Mariangela Santorsola:** formal analysis (equal), investigation (supporting), writing – review and editing (supporting). **Ernst‐Detlef Schulze:** funding acquisition (equal), writing – review and editing (equal). **Thomas Denk:** resources (equal), writing – review and editing (equal). **Daniele De Luca:** formal analysis (supporting), writing – review and editing (supporting). **Marco Cosimo Simeone:** conceptualisation (equal), funding acquisition (equal), resources (equal), supervision (lead), writing – original draft (equal).

## Conflicts of Interest

The authors declare no conflicts of interest.

## Supporting information


**File S1:** Listing the abundance of detected OTU and ASV representative sequence sets.

**File S2:** Summarising the inference difficulty scores and nonparametric bootstrap supports for (alternative) topological aspects seen in the inferred ML trees (including MOTHUR‐OTUs/‐ASVs with an abundance of ≥ 25).

**File S3:** Graphically enhanced versions of all inferred per‐sample ML trees, with subtrees representing known (Cardoni et al. 2022; Denk et al. 2024), and new (*F. hayatae*) 5S‐IGS main types collapsed (MOTHUR‐OTUs/‐ASVs with an abundance of ≥ 4).

**File S4:** A comparison of MOTHUR‐OTUs, MOTHUR‐ASVs and DADA2‐ASVs phylogenetic data inferred in *Quercus* spp., *Castanea sativa* and *Viola arvensis* .

## Data Availability

All files needed to reproduce the here reported results (scripts, OTU/ASV MSAs including a reference matrix updated with representatives of the newly found, cross‐species types, inferred trees and networks) are included in a figshare file set (Simeone et al. 2024, https://doi.org/10.6084/m9.figshare.28000349.v2). All generated raw HTS sequences are available in the NCBI Sequence Read Archive (https://www.ncbi.nlm.nih.gov/sra) under BioProjects PRJNA681175 and PRJNA1019259. All other relevant data are contained within the manuscript and its Supporting Information.
