# Supplementary material for: Pruning the Tree: Comparing OTUs and ASVs in High‐Throughput Sequencing of 5S‐IGS Nuclear Ribosomal DNA in Phylogenetic Studies
Source: Ecol Evol. 2025 Oct 7;15(10):e72242. doi: 10.1002/ece3.72242 (PMC12502049; doi:10.1002/ece3.72242)

Supplementary File S3 Partly collapsed sample-wise trees

This file includes graphically enhanced versions of the maximum likelihood trees inferred for each studied sample using the representative sequence set produced by the tested MOTHUR and DADA2 pipelines, aligned into the reference data matrix of Denk et al. (2024).

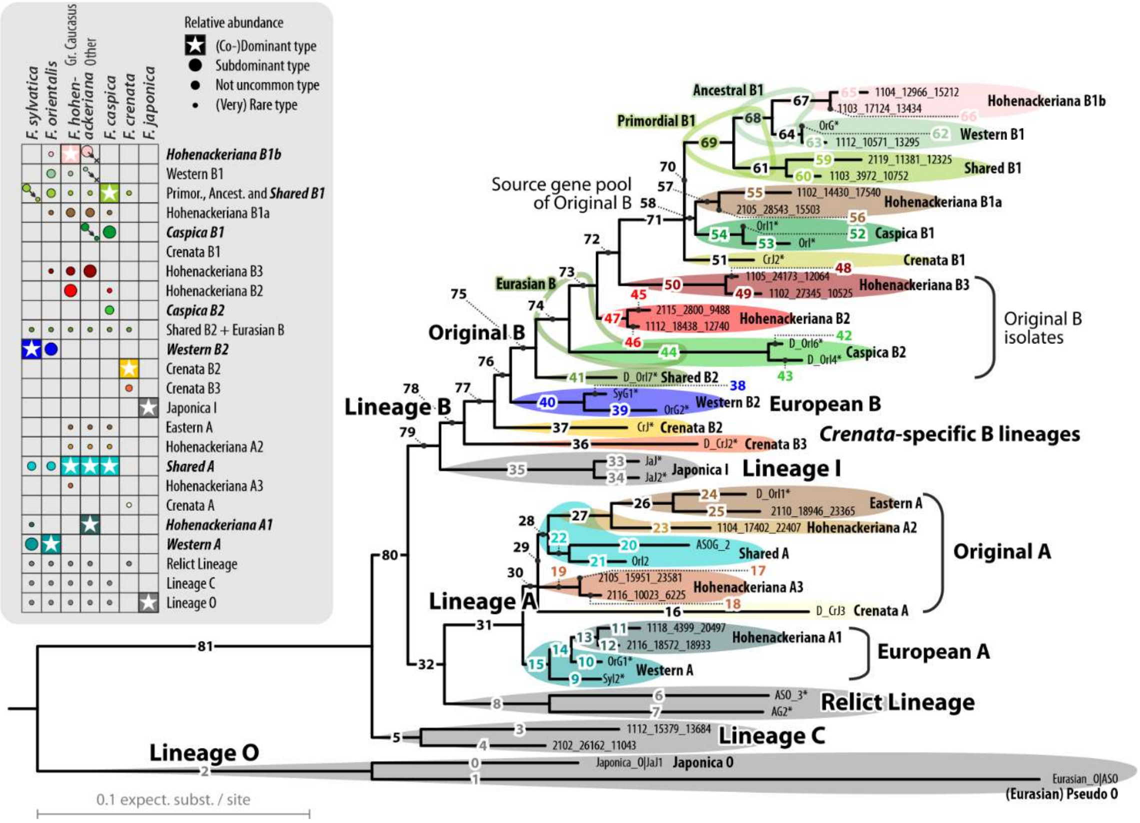

Labelling and colouration of taxonomically and phylogenetically relevant main 5S-IGS lineages and sequence types follows Denk et al. (2024), figure 6, reproduced above, with the following emendations/conventions:

- ‘**Clade B1**’ includes all B1 types, considered to represent the source gene pool of all (Eurasian) members of subgenus *Fagus*; the grade comprising the ‘Original B isolates’ and ‘European B’ (single type: **Western B2**) are collectively addressed as ‘**W. Eurasian B2/B3**’ in the graphs.
- **Primordial B1** and **Ancestral B1** are here tentatively used to refer to OTUs/ASVs not part of the clade and direct phylogenetic neighbourhood of **Western B1 + Hohenackeriana B1b** and/or **Shared B1**. In several cases, the structure of the corresponding subtree does not allow for straightforward discrimination between these sequentially very sim-

ilar types. Based on the trees inferred here, it appears that within this 'B1 core group' a further phylogenetic and taxonomic structuring can be found, so far imperfectly captured using ML tree-inference (many critical branches with diminishing bootstrap support) because of terminal noise overlying phylogenetically informative mutational patterns (e.g. in length-polymorphic sequence motives; cf. supplement to Cardoni et al. 2022, Denk et al. 2024).

- 'Original A' and 'European A' are not annotated as they are not resolved consistently as sister clades in the sample-wise trees. The reason for this insufficiency is that with increasing size of tip sets, the attraction between relatively similar variants and inclusion of aberrant, degrading variants, long-tipped variants may distort the overall topology and obscure deeper branching patterns; the alternative branches typically receive low support.
- In case of the first-time studied sample and species, *F. hayatae* sample #26, a narrow endemic of northern Taiwan, we tentatively recognize four new main types with different affinity to the main types as defined by Denk et al. (2024):
    - **Hayatae A**, a clade of Lineage A variants not found in the Western Eurasian species or *F. crenata*, placed as (low-supported) sister to **Hohenackeriana A3**, a rare type detected so far only in individuals from the Greater Caucasus (Denk et al. 2024). The **Hayatae A** variants confirm the hypothesis of Cardoni et al. (2022) that the A-B dimorphism has been inherited from the all-ancestor of (Eurasian, no data available for the N. American spp.) *Fagus* subgenus *Fagus,* nearly lost in *F. crenata*. Furthermore, A and B variants obviously underwent different sorting pathways in course of the complex speciation processes that shaped the modern-day species (see also Schulze & Grimm 2022).
    - **Hayatae B1** includes variants consistently placed as sister clade to the core clade of B1 variants found in the Western Eurasian species: **Shared B1, Western B1 + Hohenackeriana B1b**. The Evolutionary Placement Algorithm used on the reference matrix and tree of Denk et al. (2024) would classify these variants as Primordial B1 but they probably constitute a coherently distinct new type within this group, which, however, may also be present in other species as rare variants (and classified as Primordial B1 by Cardoni et al. 2022).
    - **Hayatae B2**, phylogenetically intermediate between the core clade of B1 variants and their sister clade diagnostic for the eastern species, *F: caspica* and *F. hohenackeriana* (**Caspica B1 + Hohenackeriana B1a**) may represent a first

divergence event within Clade B1. A **Hayatae B2**(-like) variant may be shared or present in the easternmost Western Eurasian species *F. caspica* (see below: **c**) but otherwise missing from all other sample-wise trees.

- **Hayatae B3**, phylogenetically most distant from the types comprising the B1 clade but less so than the B2/B3 types of the Western Eurasian species and *F. crenata*. Together with the **Hayatae B2** and even more the **Hayatae B1** variants, the phylogenetic placement of these variants imply not only early (cryptic) speciation event in the stem lineage of the Eurasian beech species but also a shared ancestry of the Taiwanese species with (part of) those of Western Eurasia that does not involve the Japanese *F. crenata* and is in stark contrast to the **Crenata B2** and **B3** variants; types representing earlier diverged lineages (near-)absent from the Western Eurasian species (one **Crenata B2** variant is shared by *F. caspica*, detected with DADA2 'pooled' and MOTHUR OTUs) but shared by *F. crenata* (as dominant type: **Crenata B2**) and *F. hayatae* (as relatively rare types).

In addition, the tip-reduction achieved using the DADA2 pipeline as alternative means to identify sets of representative sequences for each sample allowed for recognition and tracing of potentially new (typically rare) types. These isolated (pair of) tip(s) have been labelled with Latin letters (a–e when representing Lineage B variants or variants with affinity to Lineage B; y–z for Lineage A variants):

a) These tips probably represent the B↔I intermediate type recorded by Cardoni et al. (2022) and have been recorded for the *F. sylvatica, F. orientalis* and *F. hohenackeriana* samples; the three species that form the crown group of Western Eurasian beeches (cf. Schulze & Grimm 2022; Denk et al. 2024)
b) A sequentially distinct (pseudogenous?) variant with an affinity to the European B clade (comprising **Western B2** type) and shared by the western species pair *F. sylvatica*-*F. orientalis*
c) Part of the Original B clade, this long-branching tip is shared across all samples of (Eurasian) *Fagus* subgenus *Fagus*, including the newly studied sample #26 of *F. hayatae.*
d) A relatively short-branched ASV found only by the DADA2 'pooled' option for the *F. orientalis* and *F. caspica* samples, with some affinity to **Hohenackeriana B3**, a sequentially distinct, relatively rare type shared by two of the three *F. hohenackeriana* studied by Denk et al. (2024) and *F. orientalis*. Possibly a recombinant or chimeric variant mixing **Hohenackeriana B3** sequence characteristics with those of B1 types.

e) A long-branched tip unique to the *F. caspica* sample captured by three or four of the pipelines (but not included in the DADA2 'pooled' data set). It placement in the *F. caspica* trees indicates it could be a (relatively evolved) variants of the **Hayatae B2** type.

y) An extremely long-branched putative Lineage A tip found in the four of the five data sets generated for the *F. hayatae* sample, with ambiguous phylogenetic affinities, most probably representing a strongly pseudogenous or contaminant variant.

z) A relatively long-branched Lineage A tip recovered as OTU and ASV by the MOTHUR pipeline for the *F. hohenackeriana* sample. Its placement as sister to the **Hohenacker-iana A2 + Eastern A** clade appears to be genuine, indicating it could be an aberrant (pseudogenous) variant of either one of these two types.

For drafting purposes and visibility, the original trees may have been slightly modified. The unaltered trees are included in the Supporting Data Archive as raw tree output files from RAxML (NEWICK) and (preliminary) coloured and annotated NEXML-files (edited with Dendroscope). The depth of all trees has been re-scaled to the same scale, i.e. branch-lengths are directly comparable, the heights of the collapsing triangles is only relatively proportional (within the same graph) but not across graphs as the number of tips differs extremely between the DADA2 and MOTHUR approaches (see main text).

Sample 12 *F. sylvatica*

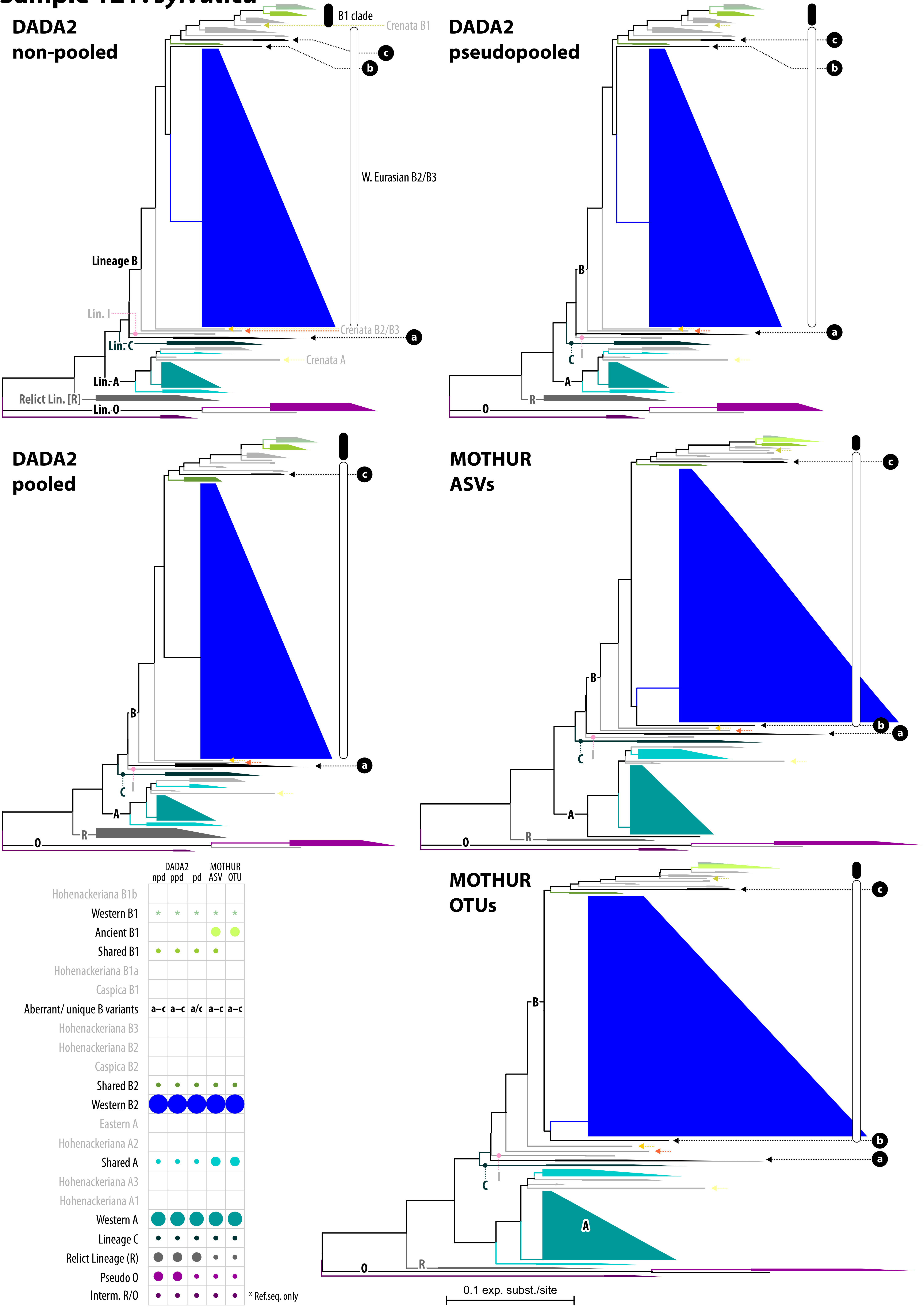

Sample 11 *F. orientalis*

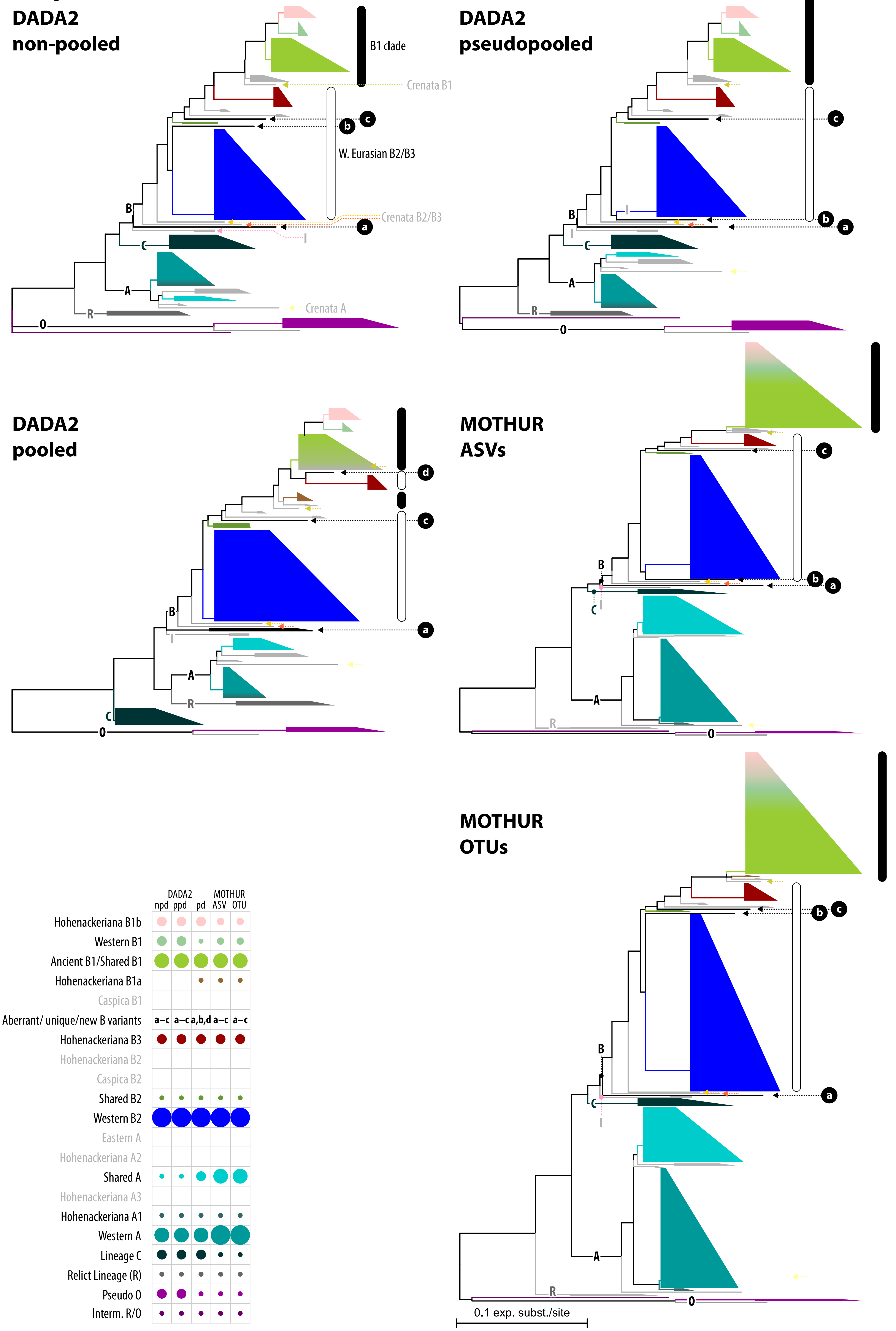

Sample 25 *F. hohenackeriana*

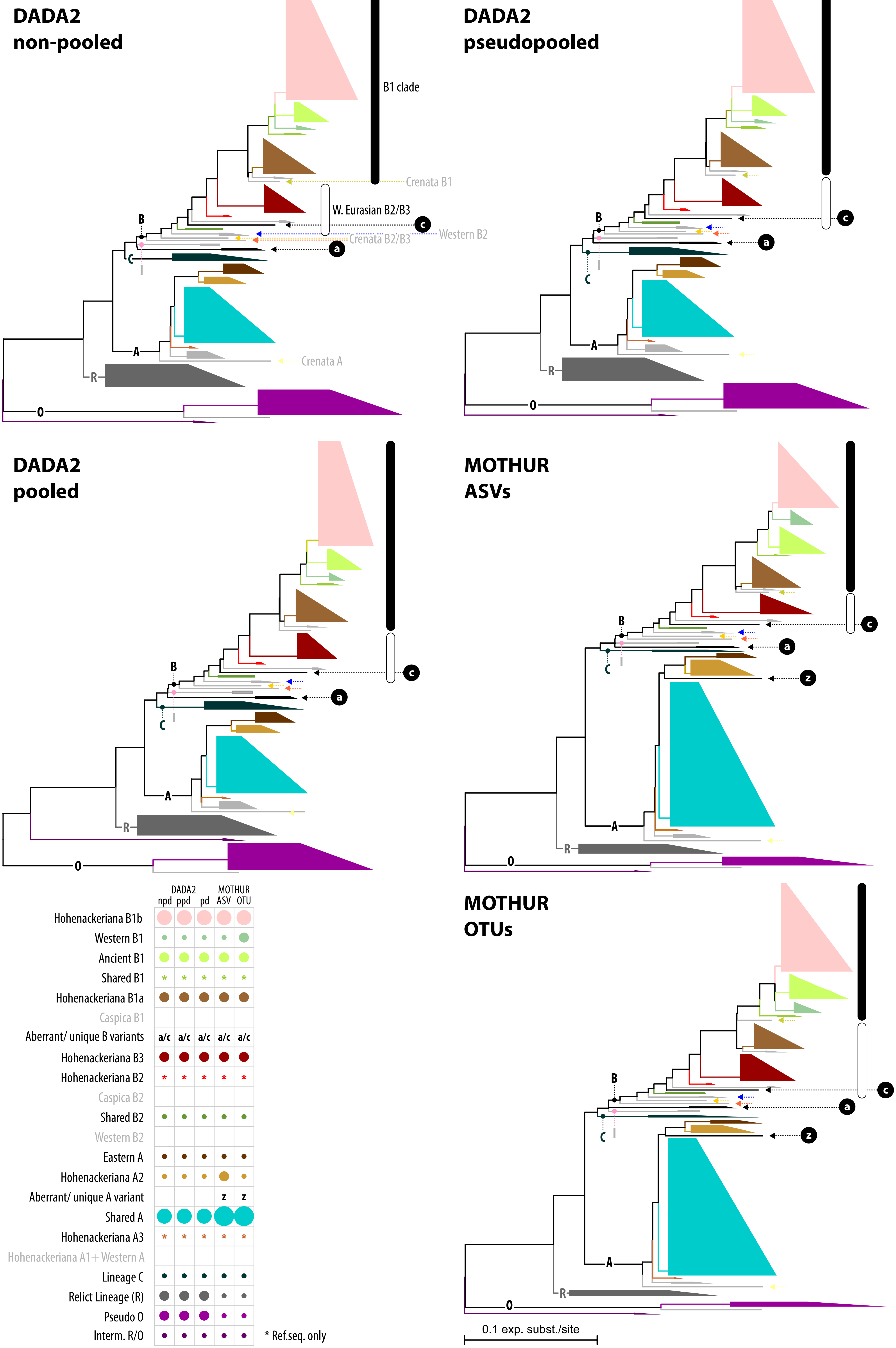

Sample 04 *F. caspica*

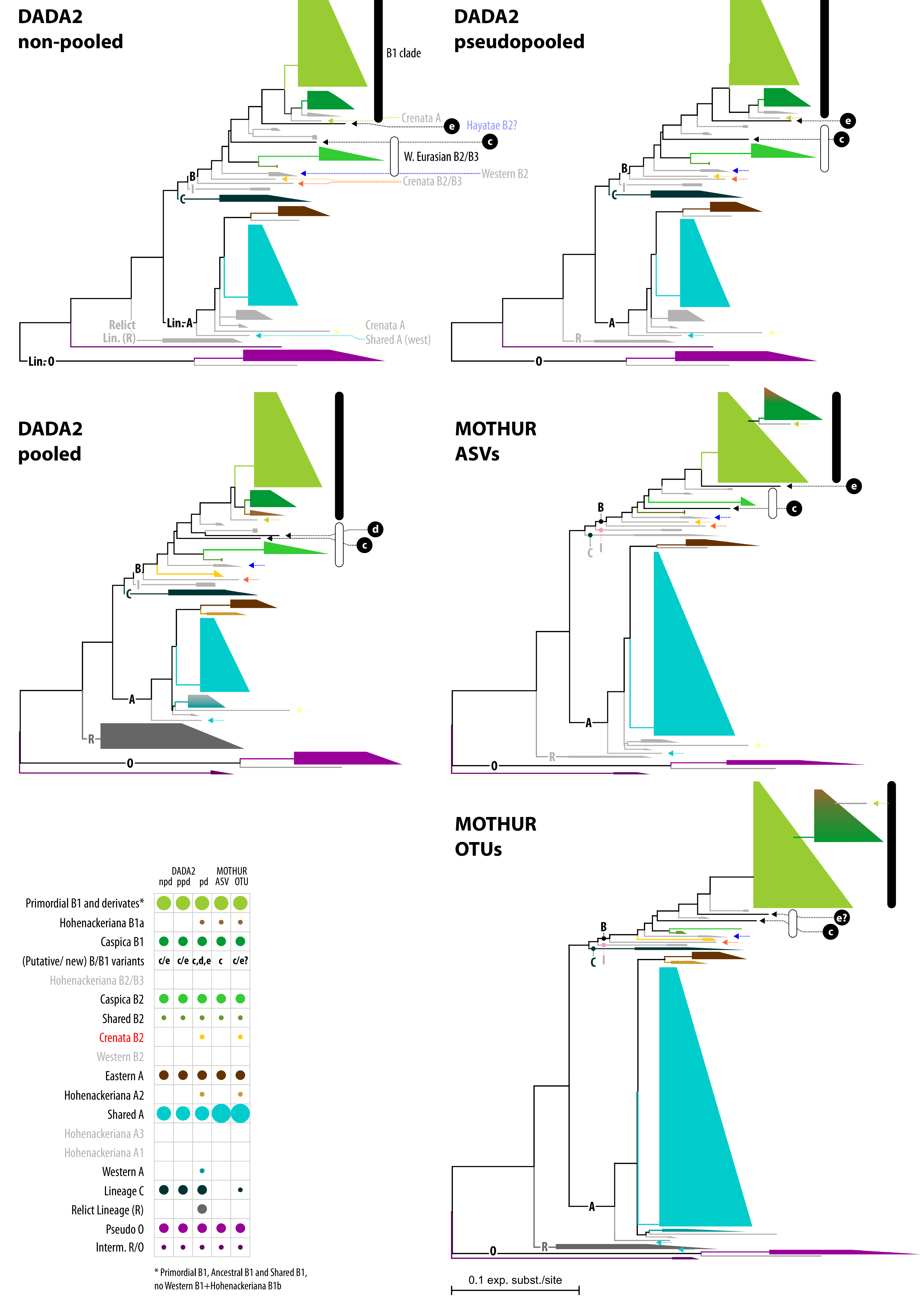

Sample 05 *F. crenata*

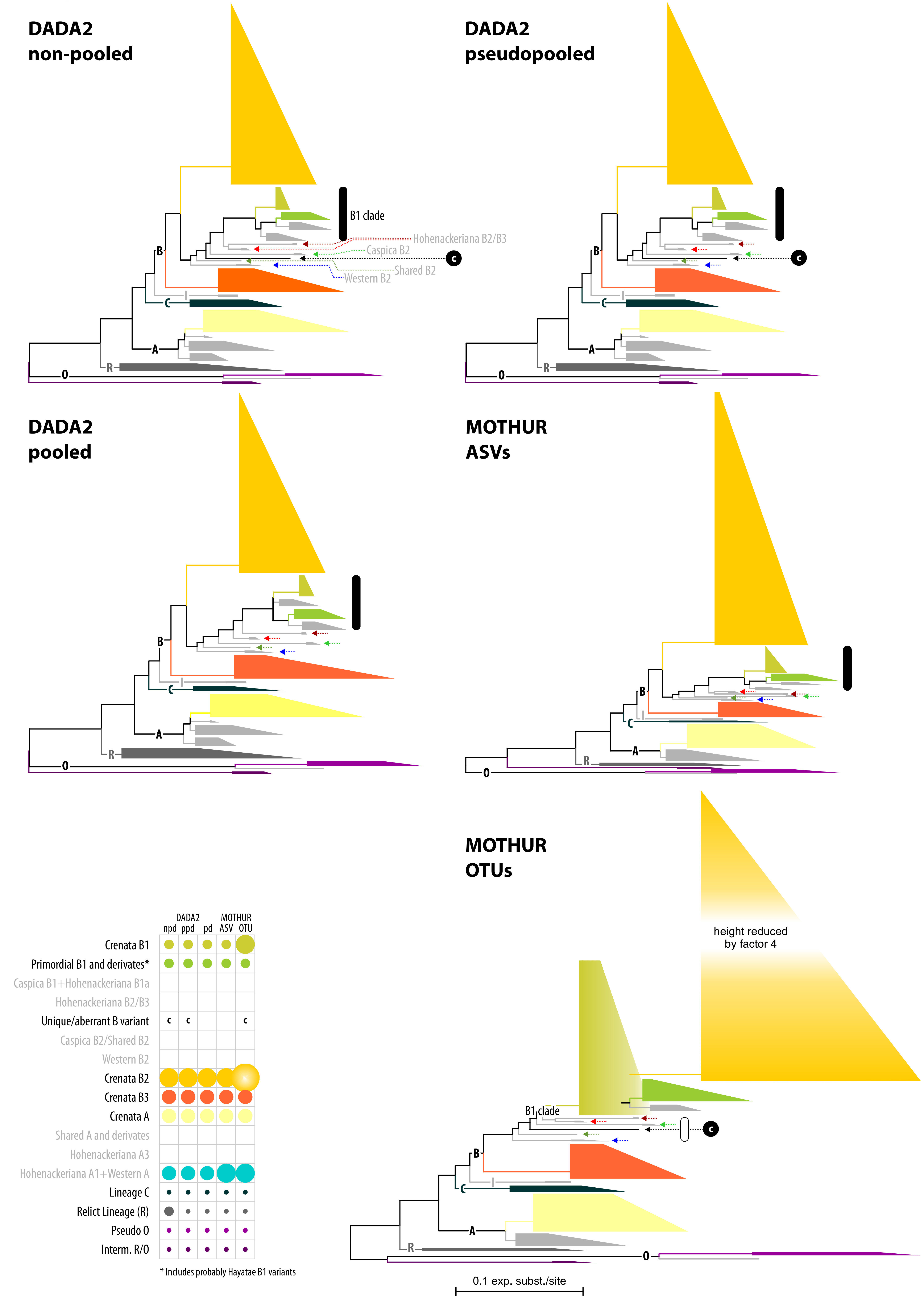

Sample 26 *F. hayatae*

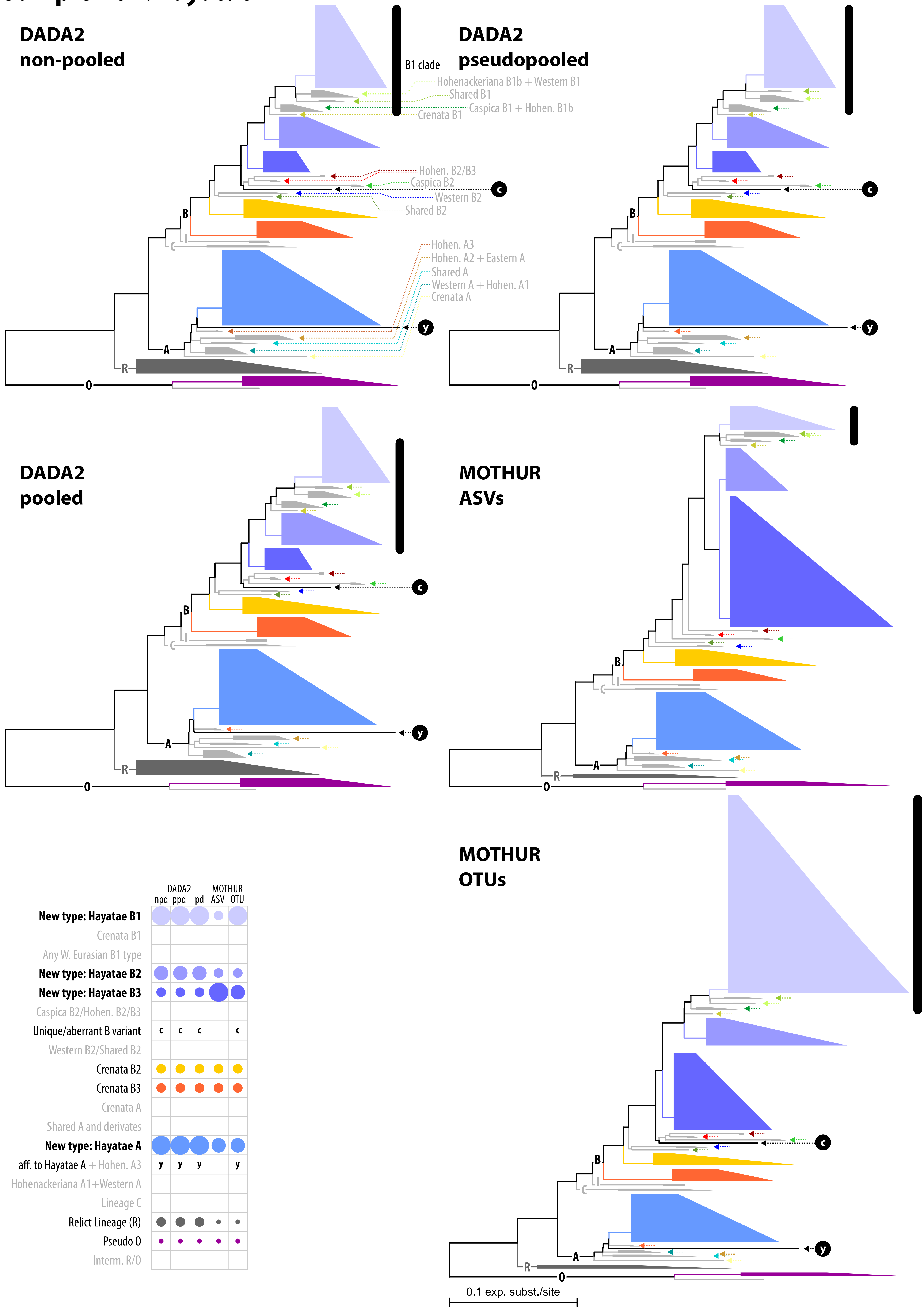

Sample 06 *F. japonica*

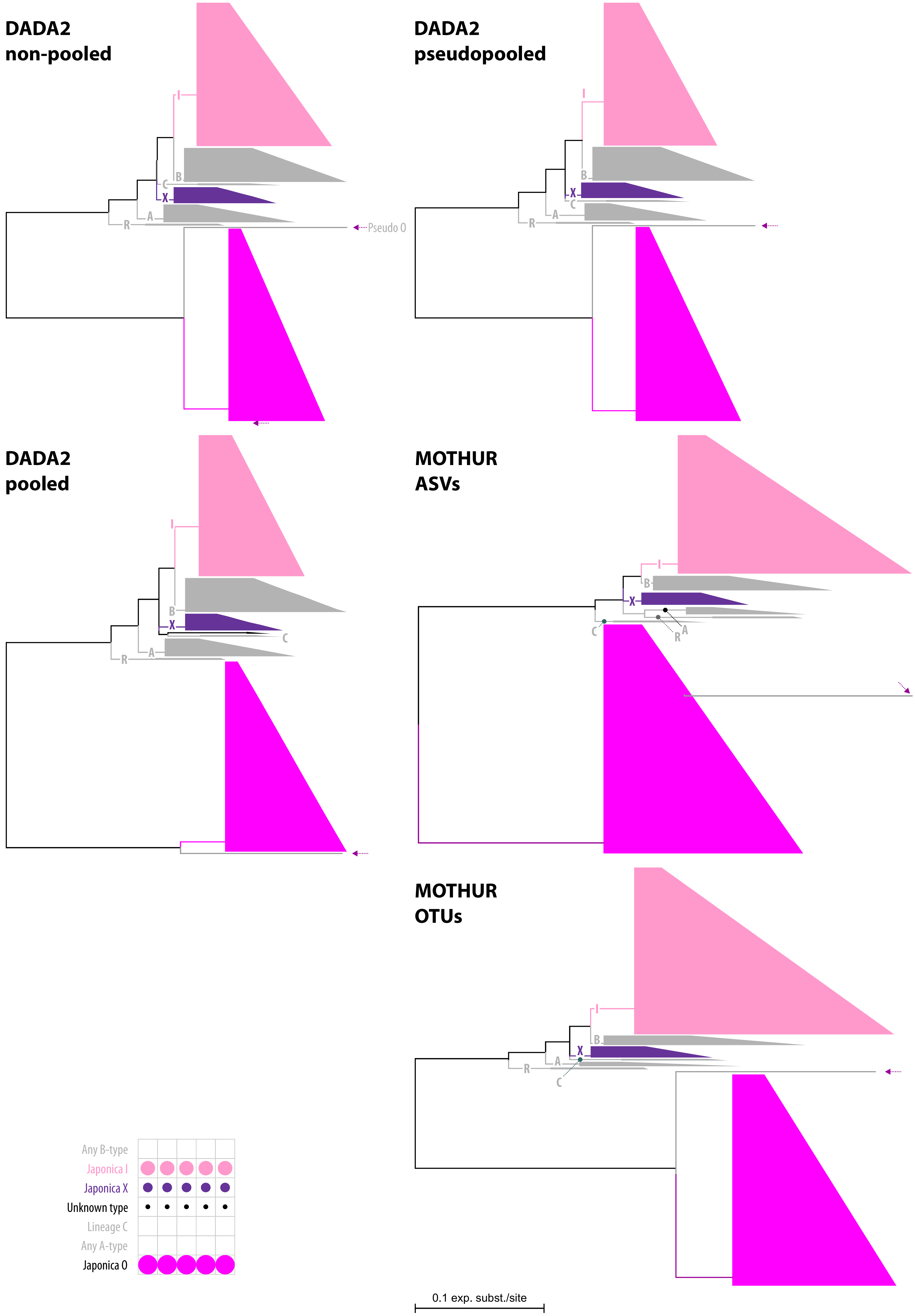

Supplement: Supplementary file 3 — File S3: Graphically enhanced versions of all inferred per‐sample ML trees, with subtrees representing known (Cardoni et al. 2022; Denk et al. 2024), and new (F. hayatae) 5S‐IGS main types collapsed (MOTHUR‐OTUs/‐ASVs with an abundance of ≥ 4). [file ECE3-15-e72242-s003.pdf]
