# Supplementary material for: Pruning the Tree: Comparing OTUs and ASVs in High‐Throughput Sequencing of 5S‐IGS Nuclear Ribosomal DNA in Phylogenetic Studies
Source: Ecol Evol. 2025 Oct 7;15(10):e72242. doi: 10.1002/ece3.72242 (PMC12502049; doi:10.1002/ece3.72242)

Supplementary file S4

In order to demonstrate the wider applicability of our findings, we here provide a comparison of MOTHUR-OTU/-ASV and DADA2-ASV (non-pooled) data obtained from three additional samples, differing in taxonomy, geographic origin, ecological context, and sample complexity.
Sample # 1 is composed of a mock community of three oak species (*Quercus*, Fagaceae) collected in North Africa (belonging to three different sections), previously investigated in Piredda et al. 2021 (sample "E5"); sample # 2 includes DNA from a single chestnut tree (*Castanea sativa,* Fagaceae) from Spain, never investigated before; sample # 3 comprises pooled DNAs from multiple individuals of an annual pansy (*Viola arvensis*, Violaceae) sampled across Italy and investigated in Scoppola et al. 2022 (sample "T10"). Data processing and phylogenetic analyses performed as reported in the main text.

Details:

Sample # 1

Individual DNA extracts: 8

Covered species: *Quercus coccifera* L. (1 individual), *Q. suber* L. (4 ind.), *Q. canariensis* Willd. (3 ind.)

Origin: Tabarka/Ain-Draham (Tunisia)

Background—The primary objective of this type of studies, consisting of mock multispecific (either known or unknown) samples, is to identify the species occurring as pooled DNAs. Taxonomic assignments of representative sequences can be done by comparison with a reference database, like e.g. NCBI GenBank (BLAST best hit), and phylogenetic tree inferences using taxonomically controlled reference data. In *Quercus*, 5S-IGS are clearly sorted by sections, with each section comprising a 5S-IGS pool notably distinct from other sections. Species assignation in *Quercus* sections *Cerris* and *Ilex* is straightforward as well, using a reference clone-sequence database of over 1000 sequences covering all Western Eurasian oak species, available in Genbank (cf. Piredda et al. 2021).

Obtained representative sequences:

268 MOTHUR-OTUs (100% identity, abundance $\geq$ 4)

173 MOTHUR-ASVs (abundance $\geq$ 4)

31 DADA2-ASVs (non-pooled)

Results—All representative sequences retrieved by the three methods were consistently assigned by BLAST to the target and/or sibling species (see Piredda et al 2021 for details on geno-taxonomic

potential resolution and bias in oaks): irrespective of the method used, the BLAST-identified sequences formed three corresponding, highly supported (94–100) clades. These clades reflect the species' sections; the only inconsistently placed sequence variant was a MOTHUR-ASV (Fig. S4-1b). MOTHUR produced an extremely high amount of highly similar OTUs and ASVs that were collected in low to high supported subclades especially in the *Q. canariensis* (sect. *Quercus*) clade. Despite the significant tip reduction, DADA2 correctly identified all species-lineages and recovered the general tree structure inferred based on MOTHUR-OTUs (Fig. S4-1d).

Conclusion—DADA2-ASV data equalled MOTHUR-OTU data in terms of precision (support of main splits), composition (sequence diversity) and sensitivity (identification of the least abundant species). Data richness was higher with MOTHUR-OTUs and -ASVs but no critical geno-taxonomic information was lost using DADA2. The DADA2 ASVs sufficiently reflect the taxonomic diversity of the sample and divergence within the species-level clades. Furthermore, the obtained DADA2 ASV dataset can be directly reused in future studies. In contrast, MOTHUR would need to be rerun again (see paper main text), and its ASVs may present some phylogenetic distortion.

Sample # 2

Single individual DNA extract

Species: *Castanea sativa* Mill.

Origin: Andalusia (Spain)

Background—No 5S nrDNA spacer sequences of *Castanea sativa* are currently available on GenBank; this sample hasn't been included in any of our prior studies either. We here assess for the first time the level of intra-genomic and intra-individual 5S-IGS diversity in chestnuts, a relative (same family: Fagaceae) of oaks (cousin lineage: *Quercus* subgenus *Cerris* share their plastome lineage with *Castanea* + *Castanopsis* and *Lithocarpus*, the Eurasian castaneoids; Zhou et al. 2022) and beeches (most distant within Fagaceae; see also Grimsson et al. 2016 for the phylo-historical background). The main objective of screening a yet unstudied taxon would be to identify the main sequence variants to develop a reference dataset and to assess the principal phylogenetic structure within and between species with as few as possible, but still representative for the overall diversity, sequences.

Obtained and used representative sequences:

2470 MOTHUR-OTUs (100% identity, abundance ≥ 4) – 464 used (abundance ≥ 25)

1643 MOTHUR-ASVs (abundance ≥ 4) – 452 used (abundance ≥ 25)

420 DADA2-ASVs (non-pooled)

To be able to quickly compare trees across methods lacking a reference data set, we dropped all MOTHUR-generated representative sequences with an abundance <25 for the phylogenetic inferences. No abundance cut-off was used for DADA2-ASVs.

Results—After applying the higher abundance cut-off for the MOTHUR-generated data, the number of representative sequences obtained with the three methods was nearly the same; analogous to what we observed for our beech data (see paper main text, Table 2). The topology of the inferred trees is visibly congruent across methods. Three major clusters are seen across all methods, two of which (red, blue) are most distinct from each other (Fig. S4-2). Overall, branch support is low (typically < 40), relating to the low overall divergence (see below). Group 1 (blue) and 3 (red in Fig. S4-2) appear to comprise many ± similar variants lacking any phylogenetic substructuring, and sister types with relatively few tips (3+ in case of Group 1, all methods; 2 in case of Group 3/MOTHUR). Group 2 (all other colours) comprising all other variants is generally more divergent and appears to be phylogenetically more sorted (several branches with BS > 40). A visible difference is that the DADA2-based tree shows two (very) rare ASV with strongly elongated terminal branches. Based on the comprehensive ML tree we selected up to 91 OTUs/ASVs representing all identified sequence groups for a first in-depth investigation of mutation patterns via a visual inspection of the placeholder alignment and inference of median networks. The (very) low branch support seen in the tree is due to a generally low compatibility and high stochasticity of most point mutations, each one supporting an internal branch of the trees. Based on the median network, we can define six basic haplotype-like groups (A to G; Fig. S4-3) that appear to be partly congruent with the groups seen in the ML trees (Fig. S4-2): all representatives of Group 1 belong to haplotype lineage E; all Group 3 representatives show haplotype B0; the pink branch within Group 2 collects lineage A haplotypes. However, the haplotypes are only semi-congruent with a conserved length-polymorphic motif in the centre of the 5S-IGS recognising three main variant groups (Fig. S4-3). An obvious advantage of DADA2 resulting from this test is that, because of its much higher reduction factor, one does not need to apply any abundance threshold to obtain a more diverse, possibly more comprehensive set of representative sequences and further reduced placeholders as basis for next analyses (Fig. S4-2c vs. Fig. S4-2a,b). However, without any abundance threshold, DADA2 can capture a higher proportion of degrading sequences (from pseudogenic/silenced arrays) that may include rare, relict types but also has a higher probability to include contaminant, strongly deviating sequences. The latter is the case for the two DADA2-ASVs with the (much) elongated tips in the ML guide tree (grey-shaded inlet in Fig. S4-2c), one belonging to a white oak (*Quercus* sect. *Quercus*), the other to Pooideae. Both were likely eliminated in the MOTHUR-generated datasets because of their limited abundance (< 25).

Conclusion—In a case like this, a recommended experimental layout (see paper main text) would be to first process the raw HTS data using DADA2 (step 1); run a quick tree-inference to identify and, when necessary, filter putative contaminant and strongly pseudogenic ASVs, i.e. ASVs producing notably elongated terminal branches (step 2); with respect to the low divergence and limited resolution encountered in this case (generally low branch support) the DADA2-obtained ASVs could be further reduced via star contraction into general (quasi-)haplotypes ("ribotypes"), and used to infer a haplotype network (e.g. Median network, minimum spanning network) to identify main types rather than inferring a comprehensive maximum likelihood tree (step 3; similar to Fig. S4-3 but further filtered for potentially pseudogenic variants); select sufficiently distinct (e.g. distinguished by >2 conserved point mutations) representative ASVs for a reference matrix and phylogenetic tree (step 4); classify MOTHUR-obtained OTUs (100% identity, abundance cutoff ≥4) into main ribotypes using e.g. the Evolutionary Placement Algorithm (EPA; Berger et al. 2011) and the DADA2-derived reference tree and matrix to search and quantify eventual peculiar/rare phylogenetic imprints and ribotypes (step 5). This process could then be extended to samples of other species of *Castanea* (or likewise structured genera) to discern between specific and shared ribotype groups, investigate the origin of enigmatic individuals (e.g., elite germplasm, artificial hybrids, long-lived and monumental trees) or gene flow among naturalized scattered and isolated populations.

Sample # 3

Individual DNA extracts: 4

Species: *Viola arvensis* Murray

Origin: Italy, various populations (details provided in Scoppola et al. 2022)

Background—*Viola arvensis* is an annual pansy, occurring near-exclusively as a weed on cultivated lands. It is a member of the allotetraploid Sect. *Melanium* Ging, Subsect. *Bracteolatae* Kupffer, together with three other, morphologically high-similar taxa, sometimes informally referred to as the '*V. tricolor*-species complex' (Scoppola et al. 2022). The chromosome base number remains unclear for the entire section, which is characterized by marked polyploidy and dysploidy, likely indicating a considerable chromosomal remodelling during its evolution, involving a highly reticulate phylogenetic history. In this type of studies, the principal objective is to identify species and sorting out dubious samples, or to derive some insights into genome origin and composition. Given the complexity of the processes involved, no other molecular markers are currently available which would allow for fast, reliable and comprehensive information on these issues.

5S-IGS amplicon variation in *V. arvensis* has been investigated in Scoppola et al. (2022) together with additional 18 congeneric samples. They found (at least) four main sequence clusters: "*Ambiguous Melanium*" (i.e., variants shared among members of the entire section), "*Ambiguous Tricolor*" (variants shared among members of the '*V. tricolor*-species complex'), "*Specific Arvensis*" (exclusive, intra-specifically shared variants), and "*Local Arvensis*" (exclusive, individual variants). Based on our previous work and limit the intricacy of the MOTHUR-generated datasets, we opted for a reduced MOTHUR-obtained sequence dataset (abundance ≥ 25) to allow an easy exploration of the phylogenetic trees. DADA2 ASVs required no abundance cutoff (as in the case of the *Castenea sativa* sample above). Given the expected complexity of the highly reticulate phylogenetic signals, sequence variation was explored with a Neighbor-Net (NNet) network.

<u>Obtained representative sequences:</u>

446 OTUs (100% identity, abundance ≥ 4) – 51 used (abundance ≥ 25)

247 MOTHUR-ASVs (with abundance ≥ 4) – 48 used (abundance ≥ 25)

31 DADA2-ASVs (non-pooled)

<u>Results</u>—The number of representative sequences obtained with the three methods is not much different. Using an abundance threshold of ≥ 25 for the MOTHUR-OTUs and ASVs, as in the case of *Fagus* (cf. main text, Table 2) and *Castanea sativa* (see above), the number of OTUs and ASVs obtained is about the same. To correlate the representative sequences across methods, we inferred a total Neighbor-Net (Fig. S4-4) and explored the congruency of the OTUs' and ASVs' placement in the clusters produced. As expected (Scoppola et al. 2022), the detected phylogenetic scenario appears very complicated, subtending the occurrence of both intra-locus and intra-genomic (multiple 5S loci) variation, likely following the already well-known auto-/allopolyploidization, hybridization, and introgression processes. Three major clusters (labelled I to III) are congruently identified by the three datasets; additional subclusters (a-c) are congruently identified by the three methods as well, but only DADA2-ASVs detected subcluster Ia and both DADA2 and MOTHUR-OTUs detected IIIb. In agreement with Scoppola et al. (2022; Fig. 5), the strongly diverging cluster I likely corresponds to the lineage comprising specific sequences of *V. Arvensis*, *V. tricolor* and further species ("*Ambiguous Melanium*"); DADA2 subcluster Ia may therefore identify relevant variation for phylogenetic/taxonomic inferences overlooked by MOTHUR datasets; this missing variation is probably contained in the low abundance MOTHUR sequences, removed with the applied abundance cut-off. Subclusters II and III appear highly reticulated and characterised by very low divergence. These subclusters group specific sequences or sequences shared among related species (informal taxonomic groups; Scoppola et al. 2022); the lower number of sequences retrieved by DADA2 however comprise all variation detected by MOTHUR (Fig. S4-5a,c) and provide a much clearer

inspection of the reticulation processes involved. Larger multispecific analyses would be needed be correctly address the identity of each cluster and subcluster; indeed, dealing with restricted, but equally informative, datasets would allow more consistent inferences. At this regard, another advantage of DADA2 is that the produced ASV dataset(s) can be directly compared across different studies, contrarily to OTUs, thus allowing a more efficient production of reference sequences and trees or networks, for progressively increasing information recovery. In conclusion, if a study's main objective is to build a reference tree and matrix that can be further expanded with growing samples, to quickly distinguish auto- and allopolyploids, and/or to assess the (reticulate) taxonomic affinities of individuals across many samples, DADA2-generated ASVs are more efficient and practical than MOTHUR-generated OTUs or ASVs with abundance $\geq$ 25. Subsequently, the Evolutionary Placement Algorithm (EPA; Berger et al. 2011) can be applied to rare OTUs to search and quantify eventual peculiar imprints with a possible (phylo)geographic significance.

Cited literature

Berger SA, Krompass D, Stamatakis A (2011) Performance, accuracy, and web server for evolutionary placement of short sequence reads under maximum likelihood. Systematic Biology, 60, 291–302. https://doi.org/10.1093/sysbi o/syr010

Denk T, Grimm GW, Manos PS, Deng M, Hipp AL (2017) An updated infrageneric classification of the oaks: Review of previous taxonomic schemes and synthesis of evolutionary patterns. In E. Gil-Pelegrín, J. Peguero-Pina, & D. Sancho-Knapik (Eds.), Oaks physiological ecology. Exploring the functional diversity of Genus *Quercus* L.. Springer, Cham. https://doi.org/10.1007/978-3-319-69099-5_2

Grímsson F, Grimm GW, Zetter R, Denk T (2016) Cretaceous and Paleogene Fagaceae from North America and Greenland: evidence for a Late Cretaceous split between *Fagus* and the remaining Fagaceae. – Acta Palaeobotanica 56: 247–305. https://doi.org/10.1515/acpa-2016-0016

Hipp AL, Manos PS, Hahn M, Avishai M, Bodénès C, Cavender-Bares J, Crowl A, Deng M, Denk T, Fitz-Gibbon S, Gailing O, Socorro González-Elizondo M, González-Rodríguez A, Grimm GW, Jiang X-L, Kremer A, Lesur I, McVay JD, Plomion C, Rodríguez-Correa H, Schulze E-D, Simeone MC, Sork VL, Valencia-Avalos S (2020) Genomic landscape of the global oak phylogeny. New Phytologist 226:1198-1212. https://doi.org/10.1111/nph.16162

Piredda R, Grimm GW, Schulze E-D, Denk T, Simeone MC (2021) High-throughput sequencing of 5S-IGS in oaks - exploring intragenomic variation and algorithms to recognize target species in pure

and mixed samples. Molecular Ecology Resources 21:495-510. https://doi.org/10.1111/1755-0998.13264

Scoppola A, Cardoni S, Marcussen T, Simeone MC (2022) Complex scenarios of reticulation, polyploidization, and species diversity within annual pansies of Subsect. *Bracteolatae* (*Viola* Sect. *Melanium*, Violaceae) in Italy: insights from 5S-IGS High-Throughput Sequencing and plastid DNA variation. Plants 11:1294. https://doi.org/10.3390/plants11101294

Zhou BF, Yuan S, Crowl AA, Liang YY, Shi Y, Chen XY, An QQ, Kang M, Manos PS, Wang B (2022) Phylogenomic analyses highlight innovation and introgression in the continental radiations of Fagaceae across the Northern Hemisphere. Nature Communications 13:1320. https://doi.org/10.1038/s41467-022-28917-1.

Figure S4-1. Circular (rooted) maximum likelihood trees for a mock-community of oaks. (a) MOTHUR-OTUs; (b) MOTHUR-ASVs; (c) DADA2-ASVs (non-pooled option). MOTHUR datasets generated with 100% identity threshold and abundance cut-off ≥ 4. Colouring represents the taxonomy of the representative reads: green, *Q. coccifera*/sect. *Ilex*; red: *Q. suber*/sect. *Cerris* (sections of subgenus *Cerris*); blue: *Q. canariensis*/sect. *Quercus* (subg. *Quercus*). Thickness reflects branch support (50-100). A rectangular phylogram of the DADA2-ASV dataset is shown in (d). All trees are systematically rooted at the subgeneric splits (Denk et al. 2017; see also Hipp et al. 2020).

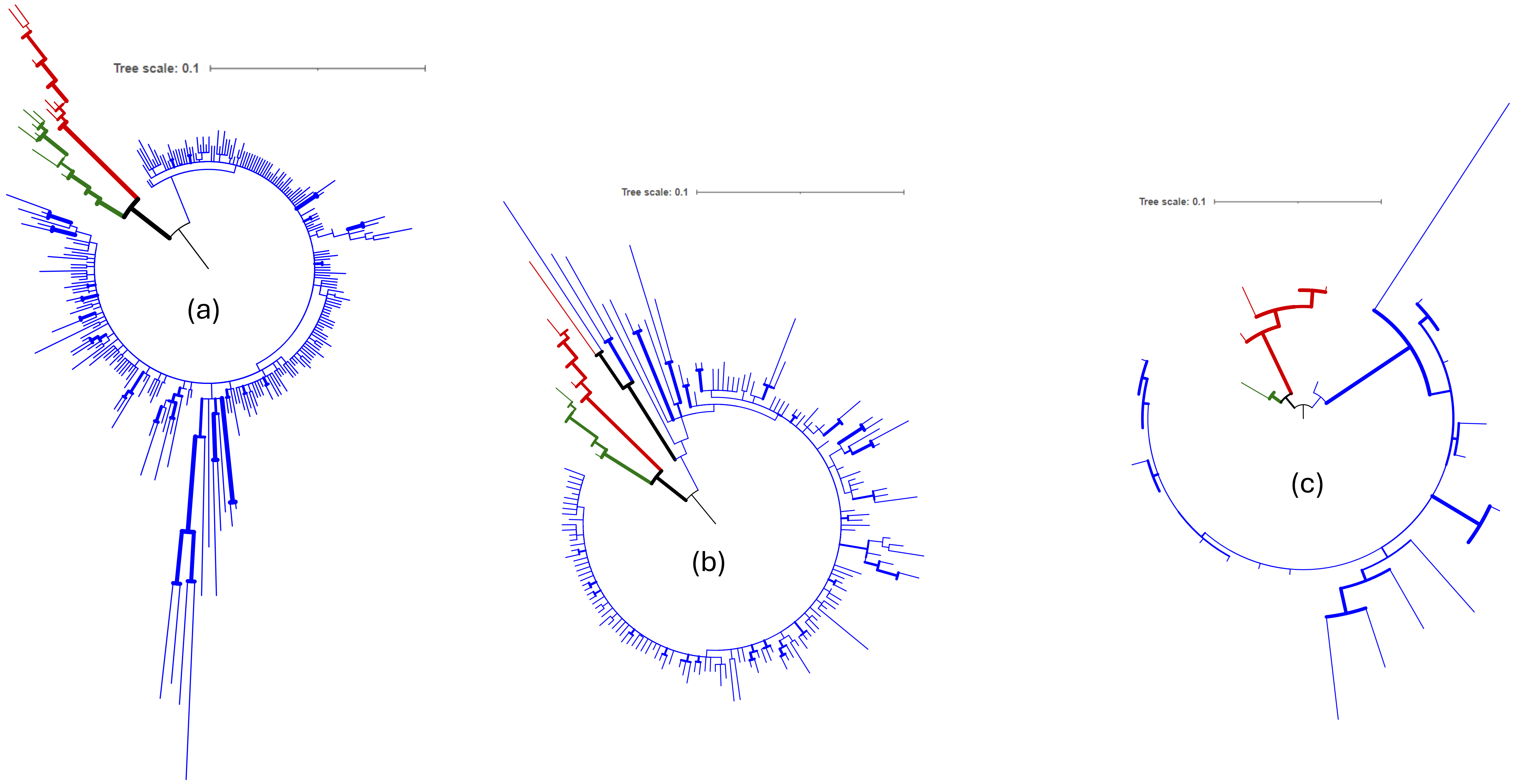

(d)

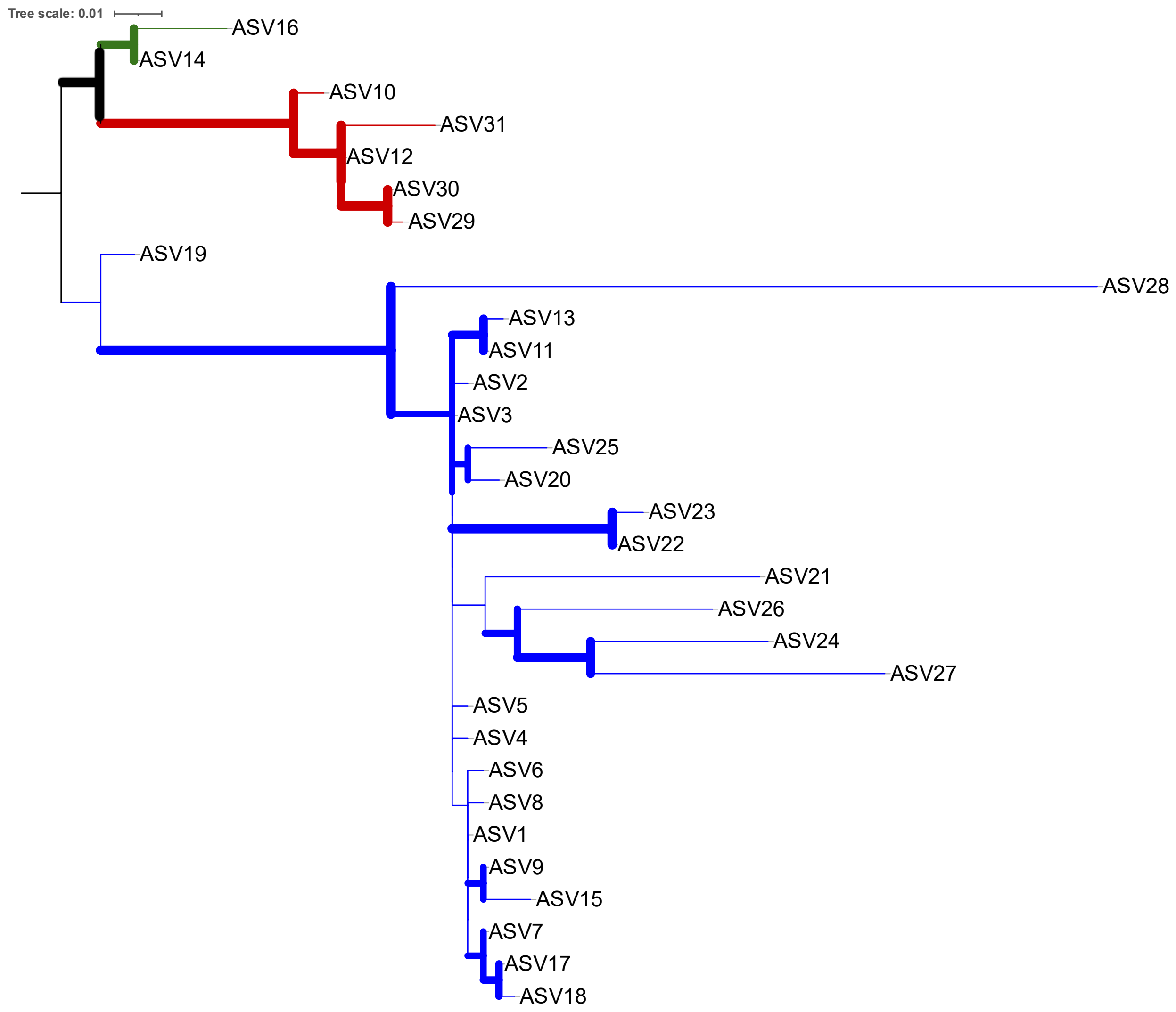
Tree scale: 0.01
ASV16
ASV14
ASV10
ASV31
ASV12
ASV30
ASV29
ASV19
ASV28
ASV13
ASV11
ASV2
ASV3
ASV25
ASV20
ASV23
ASV22
ASV21
ASV26
ASV24
ASV27
ASV5
ASV4
ASV6
ASV8
ASV1
ASV9
ASV15
ASV7
ASV17
ASV18

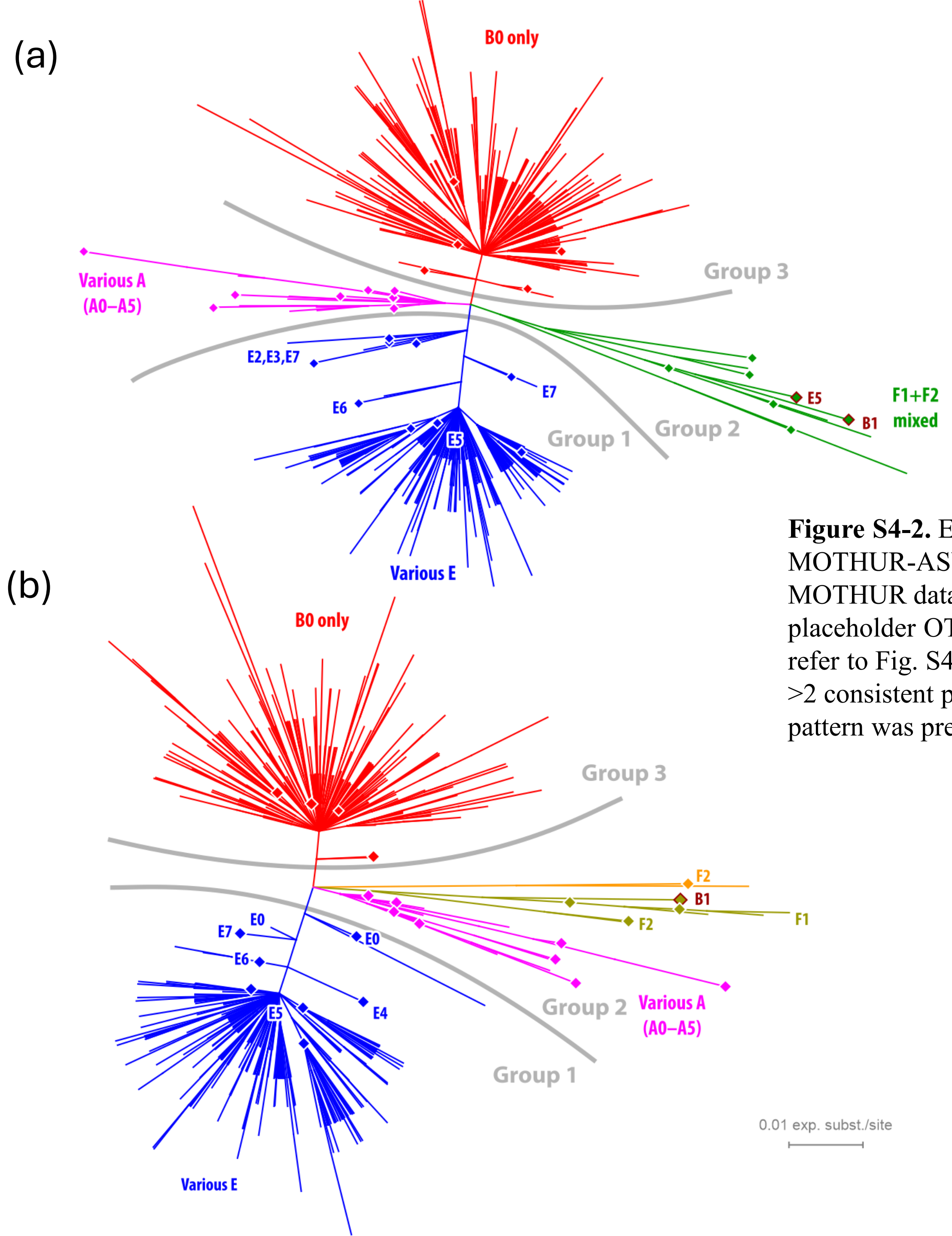

Figure S4-2. Equal-scaled radial maximum likelihood trees for the tested chestnut sample. (a) MOTHUR-OTUs; (b) MOTHUR-ASVs; (c) DADA2-ASVs, with some long-branching terminal branches reduced (see inlet, next page). MOTHUR datasets generated with 100% identity threshold and abundance cut-off ≥ 25 Diamonds represent the 91 placeholder OTUs and ASVs selected from the shown trees for a first in-depth analysis of mutation patterns, labels refer to Fig. S4-3. The red and blue groups are possibly congruent across methods (main sequence variants differing in >2 consistent point mutations), while all other-coloured groups have (partly) shifting composition (pending which site pattern was preferred during tree inference).

(c)

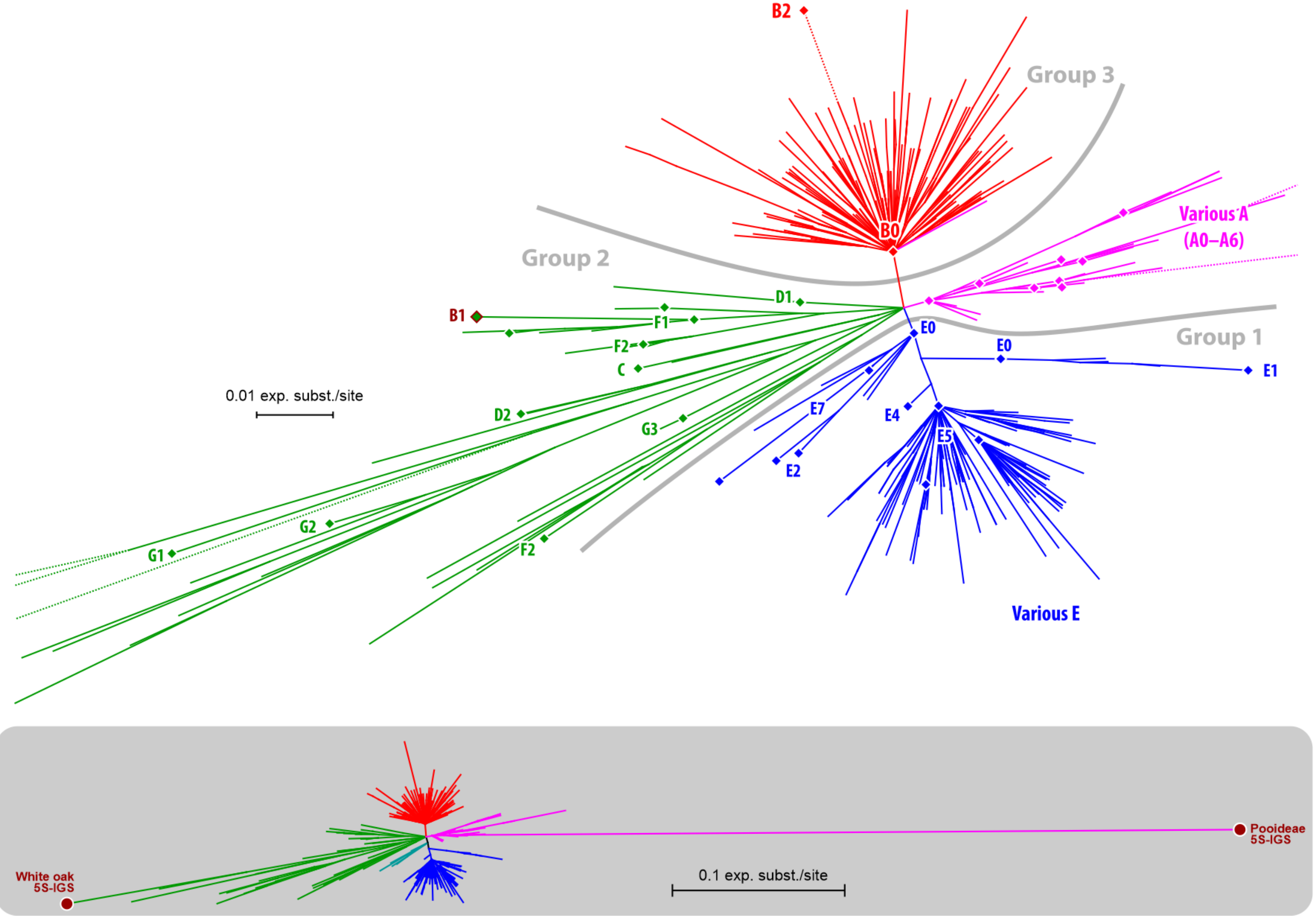

B2
Group 3
B0
Various A
(A0–A6)
Group 2
D1
B1
F1
E0
F2
E0
Group 1
C
E1
0.01 exp. subst./site
D2
E7
E4
G3
E5
E2
G2
G1
F2
Various E
White oak
5S-IGS
Pooideae
5S-IGS
0.1 exp. subst./site

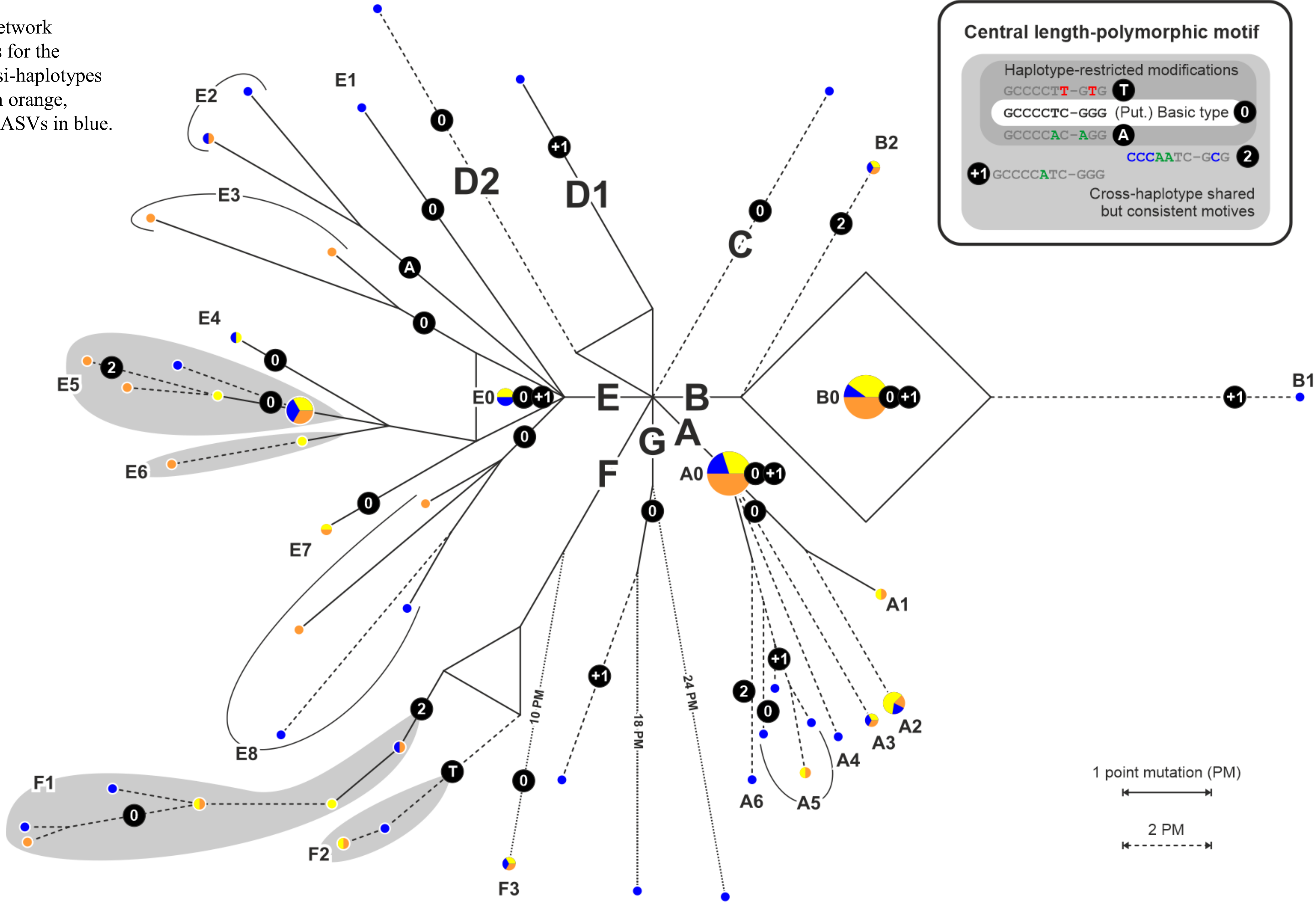

Figure S4-3. Simplified median network grouping the selected OTUs/ASVs for the *Castanea* 5S-IGS sample into quasi-haplotypes (labelled A to G). Mothur-OTUs in orange, Mothur-ASVs in yellow, DADA2-ASVs in blue.

Figure S4-4. Neighbor-Net phylogenetic network obtained with combined MOTHUR-OTUs, MOTHUR-ASVs, and DADA2-ASVs from the *Viola arvensis* sample. MOTHUR datasets generated with 100% identity threshold and abundance cut-off ≥ 25. Colours indicate clusters (I-III) and subclusters (a-c) identified by the three approaches (OTU: Mothur-OTUs; MAS: Mothur-ASVs; ASV: DADA2-ASVs).

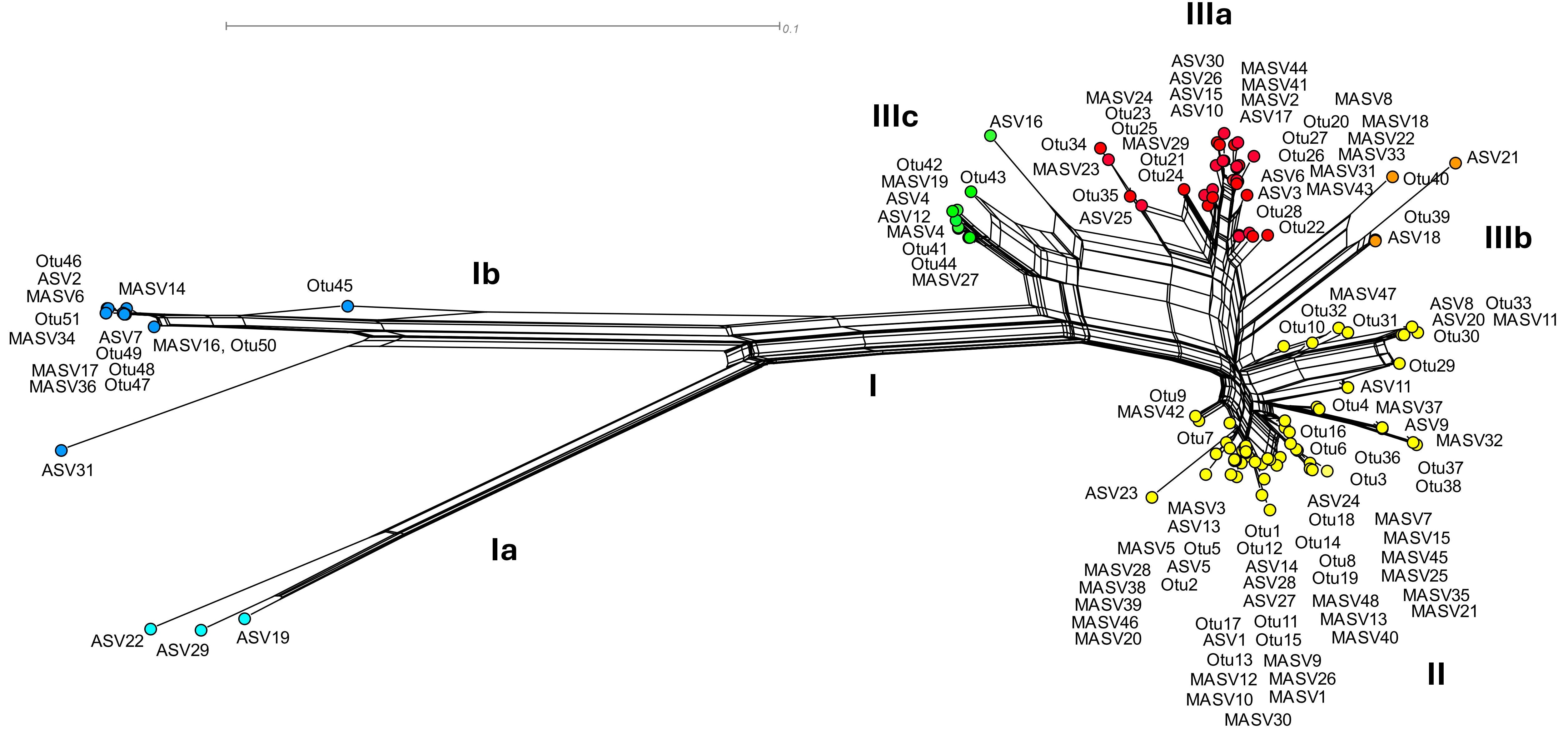

Figure S5-5. Neighbor-Net phylogenetic networks obtained with MOTHUR-OTUs (a), MOTHUR-ASVs (b), and DADA2-ASVs (c) in the *Viola arvensis* sample. MOTHUR datasets generated with 100% identity threshold and abundance cut-off ≥ 25. Colours indicate clusters and subclusters as in Fig. S4-4.

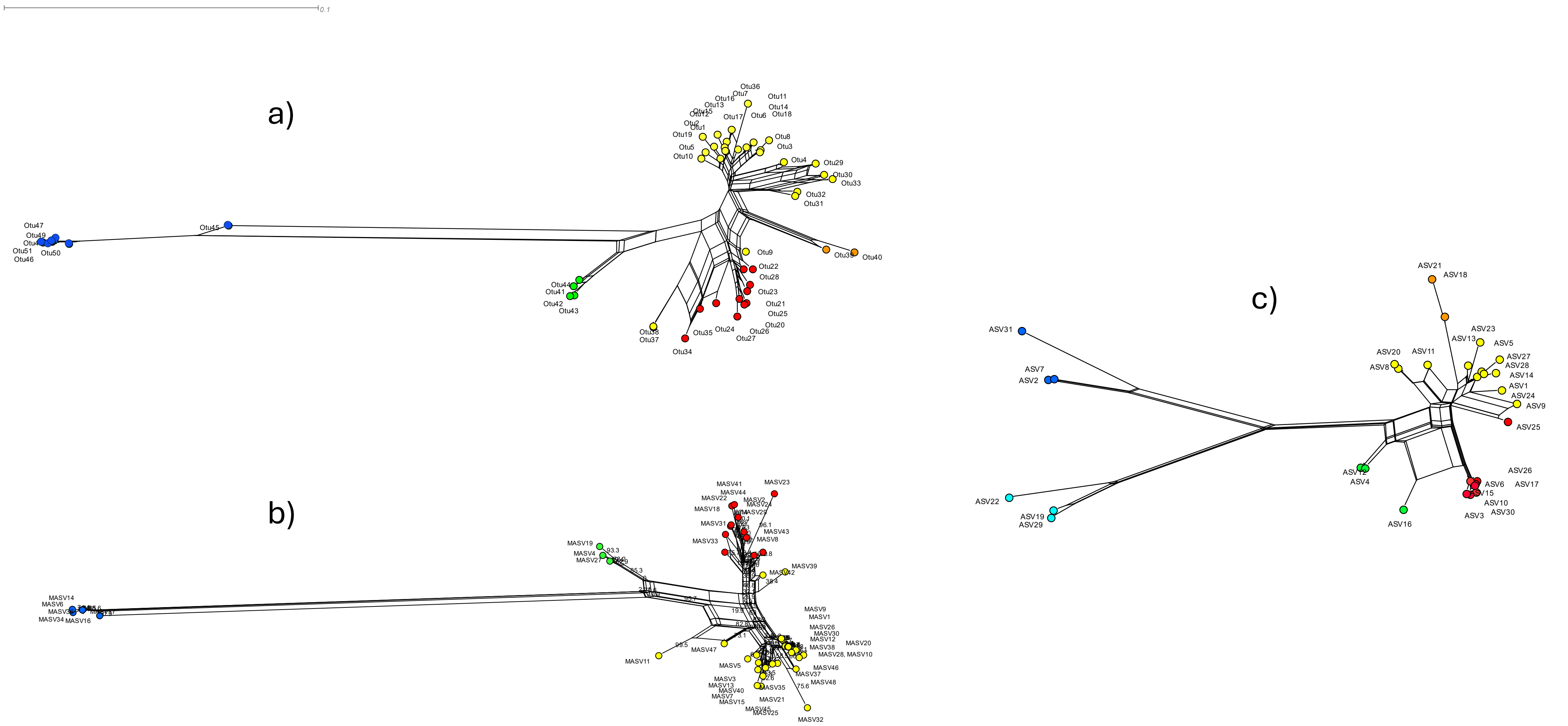

Supplement: Supplementary file 4 — File S4: A comparison of MOTHUR‐OTUs, MOTHUR‐ASVs and DADA2‐ASVs phylogenetic data inferred in Quercus spp., Castanea sativa and Viola arvensis . [file ECE3-15-e72242-s002.pdf]
